# Thyme Oil Alleviates Cadmium-Induced Disturbances in Mitotic Activity, Cytoskeletal Organization and H3T3/H3S10 Phosphorylation in *Vicia faba*

**DOI:** 10.3390/ijms27062798

**Published:** 2026-03-19

**Authors:** Natalia Gocek-Szczurtek, Mateusz Wróblewski, Aneta Żabka, Justyna T. Polit

**Affiliations:** 1 Department of Cytophysiology, Faculty of Biology and Environmental Protection, University of Lodz, 90-236 Lodz, Poland; natalia.gocek.szczurtek@biol.uni.lodz.pl (N.G.-S.); mateusz.wroblewski@biol.uni.lodz.pl (M.W.); aneta.zabka@biol.uni.lodz.pl (A.Ż.); 2 Doctoral School of Exact and Natural Sciences, University of Lodz, 90-237 Lodz, Poland

**Keywords:** mitosis, CDK, cyclin, microtubules, epigenetics, cadmium, essential oil

## Abstract

Cadmium (Cd) contamination, through induction of oxidative stress, severely impairs plant growth. Using primary roots of *Vicia faba*, we investigated how a 24 h incubation in CdCl_2_ solution (175 µM) affects mitotic progression in meristems and assessed whether thyme essential oil (TO; 0.03%, *v*/*v*), as a natural antioxidant, can protect proliferating cells during simultaneous Cd exposure. Cd strongly inhibited root growth, reduced mitotic index tenfold (to 0.6%), induced chromatin condensation, decreased CDKA protein levels and CycB transcripts and proteins, caused pronounced microtubule bundling and alterations in their arrangement, disorganization of actin filaments, and disturbances in histone H3 phosphorylation (H3T3Ph, H3S10Ph). TO led to a partial recovery of mitotic index (to ~50% of the control), normalization of chromosome condensation, maintenance of cell-cycle regulators at near-control levels, preservation of proper cytoskeletal organization, and restoration of the correct H3 phosphorylation pattern. This enabled cells to progress from metaphase to anaphase and maintain phase proportions close to the control, resulting in normal root growth. These findings indicate that TO protects the mitotic cellular environment against Cd-induced disturbances. To the best of our knowledge, this is the first evidence that TO safeguards the plant mitotic apparatus under Cd stress, highlighting its potential as a natural bioprotective agent supporting plant growth.

## 1. Introduction

Cadmium (Cd) is a major environmental pollutant that, even at low concentrations, disrupts plant physiology and poses risks to human and animal health [1,2]. Cd exposure induces multiple cellular disturbances, including oxidative stress, membrane damage, and genome instability [3,4,5]. A key mechanism of its toxicity involves interference with essential ions and proteins: Cd competes with Ca^2+^, Zn^2+^, Mg^2+^, and Fe^2+^ and binds to protein thiol groups, leading to enzyme inactivation and metabolic dysregulation [6,7]. Rapidly proliferating cells, such as plant meristematic cells, are particularly sensitive to these effects because their proper function requires strict control of genome stability and cell cycle progression [3,8,9]. Due to its direct contact with contaminated substrates, the root apical meristem represents a useful model for studying cadmium-induced alterations in mitosis.

The plant cell cycle is regulated by two principal control points (PCP1 and PCP2) located in G1 and G2 phases that respond to environmental signals [10,11,12], and its progression is controlled by cyclin-dependent kinases (CDKs) and their cyclin partners (Cyc), whose activity is further modulated by regulatory proteins, including cyclin-dependent kinase inhibitors (CKIs), that can arrest S- and M-phase CDK complexes under stress conditions [13,14,15,16,17]. Mitotic entry is driven by CycA- and CycB-dependent activation of CDKA (the plant homolog of Cdc2/CDK1) and plant-specific CDKB kinases, which together constitute the plant M-phase promoting factor (MPF) activity [18]. MPF activity is negatively regulated by WEE1-dependent inhibitory phosphorylation and activated by CDK-activating kinases (CAKs), together with removal of the inhibitory phosphate by a CDC25-like protein [19,20,21]. The active CDK–Cyc complex initiates mitosis via phosphorylation of downstream substrates, whereas mitotic exit requires degradation of mitotic cyclins mediated by the anaphase-promoting complex/cyclosome (APC/C) with the involvement of CCS52 [22,23].

The proper progression of plant cell division depends on the dynamic cytoskeleton composed of microtubules and actin filaments [24]. Microtubules, formed by α- and β-tubulin heterodimers, exhibit dynamic instability, enabling rapid reorganization during the cell cycle [25]. During interphase, cortical microtubules reorganize into the preprophase band (PPB), marking the future division site before disappearing prior to metaphase [26,27]. Concurrently, actin filaments form an “actin cage” that stabilizes the mitotic spindle and maintains its position [24]. In prophase and prometaphase, a bipolar spindle assembles despite the absence of centrosomes [28]. Spindle microtubules attach to chromosome centromeres via kinetochores, ensuring accurate chromosome segregation [29,30,31]. Improper attachment activates the spindle assembly checkpoint (SAC), which arrests the metaphase-anaphase transition by inhibiting APC/C [32]. Microtubule nucleation involves the γ-TuRC complex associated with the nuclear envelope and recruited to existing microtubules via augmin, whereas microtubule dynamics are regulated by microtubule-associated proteins (MAPs) [33,34,35,36]. In metaphase, chromosomes align at the equatorial plate through kinesin-driven activity [36,37], followed by separase-mediated cohesin cleavage and poleward chromatid separation during anaphase [36,38]. In telophase and cytokinesis, microtubules form the phragmoplast, stabilized by MAP65-3 and supported by actin filaments, guiding vesicles with cell wall components to complete cell plate formation [39,40,41]. Coordinated microtubule and actin dynamics thus ensure accurate division and daughter cell integrity [42].

During mitotic entry, epigenetic histone modifications drive chromatin condensation and centromere formation for kinetochore assembly [43,44]. These modifications also modulate sister chromatid cohesion by influencing cohesin binding and integrate environmental and cellular signals that shape chromatin states during the cell cycle [45,46]. Among the best-characterized mitotic marks are phosphorylations of histone H3 at threonine 3 (H3T3Ph) and serine 10 (H3S10Ph), which correlate with chromosome condensation [47]. H3T3 phosphorylation, catalyzed by Haspin kinase and distributed along plant chromosomes, promotes recruitment of chromosomal passenger complex (CPC) components involved in correcting kinetochore–microtubule attachments [48,49,50]. A core CPC component, Aurora B kinase, mediates H3S10 phosphorylation. In *Arabidopsis thaliana*, Aurora kinases (AtAUR1–3) phosphorylate H3S10 in pericentromeric regions, supporting cohesion maintenance and proper chromatid segregation [51,52]. The dynamics of these modifications are controlled by the balance between kinase and phosphatase activities, particularly protein phosphatases PP1 and PP2A, which regulate the phosphorylation status of mitotic proteins, chromatin and kinetochore components, thereby influencing the stability of kinetochore–microtubule attachments and the proper progression of mitosis [53].

Dysregulation of cell division control leads to genome instability, developmental defects, and reduced adaptive capacity in plants, and these disturbances are further intensified by environmental stresses associated with ecosystem degradation [23]. In the context of increasing heavy metal contamination driven by industrialization, intensive agriculture, and urbanization [54], identifying compounds that help maintain cellular homeostasis and the stability of cell division processes has become particularly important. Essential oils have attracted attention as biologically active mixtures of volatile plant metabolites. Among them, thyme essential oil (TO), rich in phenolic monoterpenes such as thymol and carvacrol, exhibits strong antioxidant properties and can modulate endogenous plant defense systems involved in redox regulation [55,56,57].

Although the protective effects of TO under cadmium stress have been reported in animal models [58], its impact on proliferating plant cells remains poorly understood. Our previous studies on root meristems of *Vicia faba* var. *minor* demonstrated that TO mitigates cadmium-induced oxidative stress in interphase cells, reduces replication stress and DNA damage, and stabilizes epigenetic modifications of histones and DNA, thereby supporting transcriptional activity [59,60].

The aim of the present study was to evaluate the protective potential of TO on mitotic cells exposed to cadmium chloride (CdCl_2_). The same experimental model as in our previous studies was employed, namely the root meristems of *V. faba*, a species widely used in plant mitosis research due to its low chromosome number (2*n* = 12), large chromosome size, and distinct morphology, features that allow precise assessment of cell division processes. Seedlings were subjected to a 24 h incubation in CdCl_2_ solution, in the emulsified form of TO, and in a combination of both substances. Seedlings incubated for 24 h in water or in the emulsifier (mixture of C14-18 and unsaturated C16-18—mono- and di-ethoxylated glycerides and ethoxylated *Brassica napus* oil) solution used to prepare the oil served as control material.

Based on literature reports indicating that cadmium interferes with cell-cycle regulation and mitotic progression in plant cells [3,61,62,63,64], we hypothesized that cadmium may disrupt the activation of mitosis by interfering with the function of the plant MPF complex, which is responsible for its initiation, and may further impair the progression of mitosis by affecting the cytoskeletal structures and mitotic chromosomes, as well as their mutual interactions. In this context, TO may exert a protective effect by safeguarding these fundamental processes in dividing cells from the toxic impact of cadmium, potentially through mechanisms related to its previously reported antioxidant properties. To test this hypothesis, we assessed primary root growth, the mitotic and phase indices, levels of CDKA and CycB, the organization of microtubules and actin filaments, as well as epigenetic modifications of histone H3 (H3T3Ph and H3S10Ph). This approach provides mechanistic insight into the effects of cadmium on plant cells and allows evaluation of the protective potential of TO under heavy metal-induced stress.

## 2. Results

Prior to the main study, a preliminary screening of Cd (100–200 µM) and TO (0.01–0.06% *v*/*v*) concentrations, including their combinations, was conducted based on the mitotic index in root meristem cells (Table S1, Supplementary Materials). For the main study, 175 µM Cd was selected as a concentration that strongly (but not extremely) inhibited the mitotic index, and 0.03% TO was chosen as the lowest dose consistently eliciting a protective effect without markedly affecting cell division when applied alone.

### 2.1. Primary Root Growth

**Figure 1:**
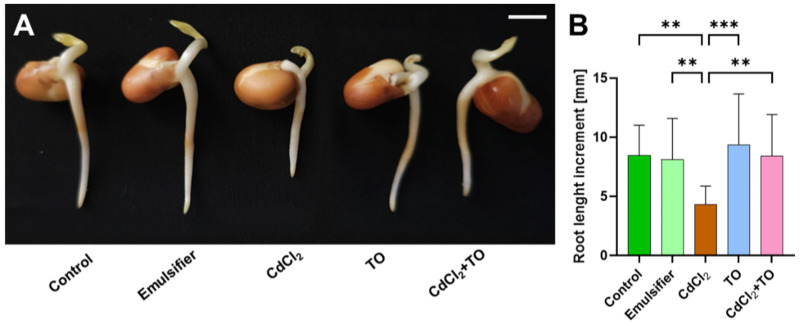
Seedlings of *V. faba* (**A**) and increments in primary root length (**B**) after 24 h of incubation in water—Control, emulsifier, CdCl_2_, thyme essential oil (TO), and the combination of CdCl_2_ and TO. Scale bar = 10 mm. Data are presented as mean ± SEM *(n* = 17; each replicate representing an individual seedling). Statistical significance: *** *p* < 0.001; ** *p* < 0.01 (one-way ANOVA followed by Tukey’s multiple comparison test).

Primary root growth is driven by the apically located meristematic tissue. Therefore, to evaluate the effects of the tested compounds on seedling growth after 24 h of incubation, the increase in root length was analyzed (Figure 1A,B). Under control conditions, including incubation of seedlings in distilled water as well as in the emulsifier solution used for TO emulsification, embryonic roots elongated by approximately 8 mm. In the presence of cadmium (CdCl_2_), root elongation was reduced by about 50% and reached an average of 4 mm. Application of TO alone did not cause statistically significant changes in root length increment compared to the control. In contrast, simultaneous application of TO and CdCl_2_ resulted in a root length increase comparable to that observed in the control variant, despite the presence of the heavy metal.

### 2.2. Chromatin Structure and Mitotic Activity

**Figure 2:**
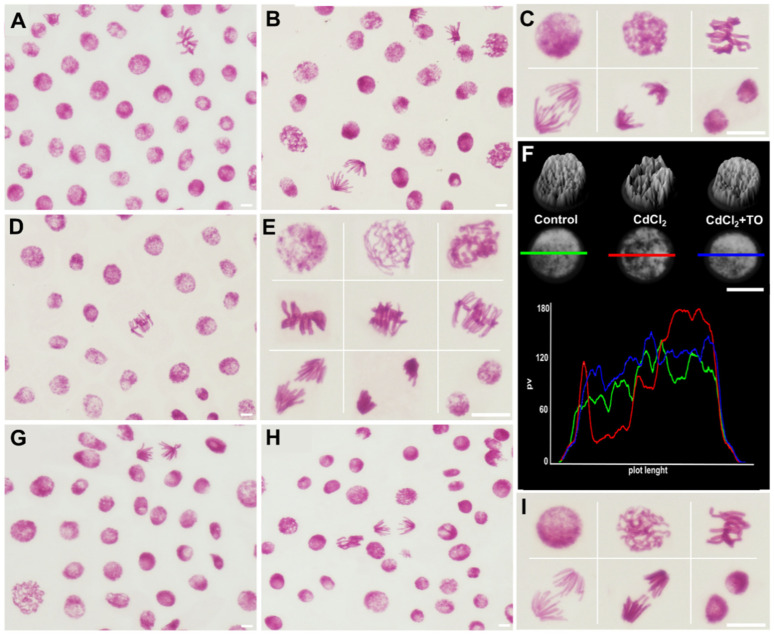
Feulgen DNA staining illustrating overall view of cell population in primary root meristems of *V. faba* after 24 h of incubation in water—Control (**A**), emulsifier (**B**,**C**), CdCl_2_ (**D**,**E**), thyme essential oil (TO) (**G**), and the combination of CdCl_2_ and TO (**H**,**I**). Higher-magnification images showing representative interphase nuclei and successive mitotic stages: control or emulsifier (**C**), CdCl_2_ (**E**), and TO or CdCl_2_ + TO (**I**). Identical mitotic figures for control and emulsifier, as well as for TO and CdCl_2_ + TO, are shown once due to the lack of detectable structural differences. (**F**) Densitometric plots illustrating relative chromatin condensation (expressed as pixel values (pv) from 1 to 255) in nuclei from control (green line), CdCl_2_-treated (red line), and CdCl_2_ + TO-treated (blue line) meristems. Plot length = 18 µm. Scale bar = 10 µm.

**Figure 3:**
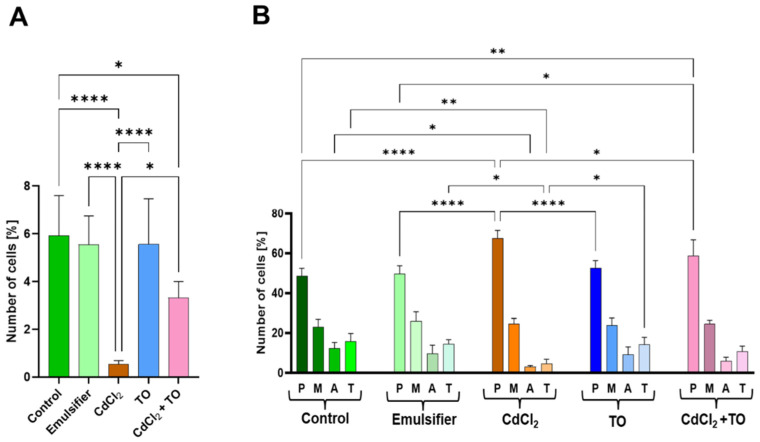
Mitotic index (% ± SD) (**A**) and mitotic phase indices (% ± SD) (**B**) in primary root meristem cells of *V. faba* after 24 h of incubation in water—Control, emulsifier, CdCl_2_, thyme essential oil (TO), and a combination of CdCl_2_ and TO. Abbreviations: P—prophase; M—metaphase; A—anaphase; T—telophase. Mean values of the mitotic index were calculated based on five root meristems, with 500 cells analyzed per meristem. Statistical significance: **** *p* < 0.0001, ** *p* < 0.01, and * *p* < 0.05 (one-way ANOVA followed by Tukey’s multiple comparison test).

Differences in the intensity of primary root growth observed among the experimental variants prompted an analysis of meristematic cell activity (Figure 2 and Figure 3). In control and emulsifier-treated meristems, interphase nuclei stained with the Feulgen method displayed a typical reticulate nuclear structure with visible chromocenters (Figure 2A–C). Within the uniformly stained chromatin, more highly condensed regions were distinguishable, forming characteristic chromocenters corresponding to heterochromatin-rich pericentromeric and telomeric regions. Chromatin organized in this manner underwent normal condensation into mitotic chromosomes, and the resulting mitotic figures exhibited typical chromosome morphology (Figure 2C). Under these conditions, the mitotic index reached approximately 6%. Among dividing cells, prophase predominated (about 50%), followed by metaphase (up to 25%). Anaphases and telophases accounted for over 10% and about 15% of all mitotic figures, respectively (Figure 3A,B).

In primary root meristems exposed to cadmium, interphase nuclei exhibited a pronounced change in chromatin structure (Figure 2D,E), as confirmed by both 3D surface profiles and densitometric analysis of Feulgen-stained nuclei (Figure 2F). Chromatin appeared less uniform and displayed a network-like, lacy pattern, with DNA-rich regions interspersed with unstained or lightly stained gaps (sharply defined valleys and high peaks in the 3D images; Figure 2F). Chromocenters, clearly visible and not obscured by the surrounding chromatin, were particularly distinct and exhibited increased compaction (Figure 2D–F). The interphase chromatin subsequently condensed into mitotic chromosomes, which in early prophase retained this lacy organization, became strongly and irregularly condensed in late prophase, and appeared short and highly condensed in metaphase, with telomeric regions showing a tendency to adhere. Chromatid separation during anaphase was impeded, particularly in early anaphase, due to sticky telomeric ends. These defects resulted in strongly condensed telophase and post-mitotic nuclei, whose chromatin structure resembled that of cells entering mitosis (Figure 2E). Mitotic activity was markedly reduced, not exceeding 0.6%. Among dividing cells, over 65% were in prophase, approximately 25% in metaphase, and cells in later stages (anaphase and telophase) collectively accounted for less than 10% (Figure 3A,B).

The emulsified form of TO did not induce noticeable changes in the structure of interphase nuclei or mitotic chromosomes compared with the control (Figure 2G), nor did it significantly affect the mitotic index or the proportions of cells in different mitotic phases (Figure 3A,B). Simultaneous treatment with TO and cadmium mitigated severe structural alterations: interphase nuclei and their densitometric profiles showed only a slight shift toward increased condensation, remaining clearly similar to the control (Figure 2H,I,F). Mitotic chromosome condensation and mitotic figure abnormalities were also markedly reduced (Figure 2I). This was accompanied by partial preservation of mitotic activity, exceeding 3%, and smaller deviations in the distribution of cells across mitotic phases compared with the control (Figure 3A,B).

### 2.3. Dynamics of CDKA Kinase and CycB

The observed decrease in mitotic activity in primary root meristems of *V. faba* under cadmium exposure prompted an analysis of principal mitotic regulators, including CDKA, which remains relatively constant throughout the cell cycle, and its regulatory partner, CycB, characterized by periodic synthesis and degradation [65].

**Figure 4:**
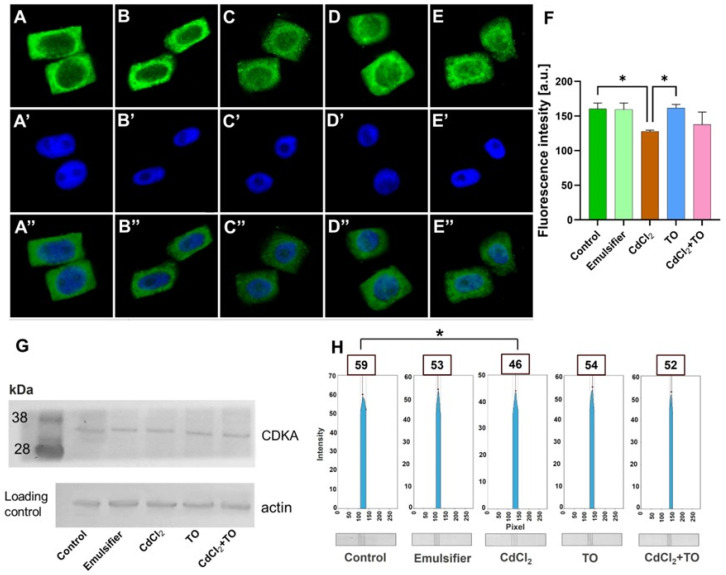
Immunofluorescence detection of CDKA in primary root meristem cells of *V. faba* after 24 h of incubation in water—Control (**A**), emulsifier (**B**), CdCl_2_ (**C**), thyme essential oil TO (**D**) and a combination of CdCl_2_ and TO (**E**). Corresponding images of cell nuclei stained with DAPI (**A′**–**E′**), and merged images (**A″**–**E″**). Scale bar = 10 µm. Median (±CI; *n* = 5 biological replicates; each representing an individual seedling) intensity of CDKA labeling; approximately 100 cells were analyzed per treatment (**F**). Statistical significance: * *p* < 0.05 (Kruskal–Wallis test with post hoc Dunn’s multiple comparison test). Western blot analysis of CDKA in protein extracts obtained from the apical region of primary roots after 24 h incubation under the same treatment conditions (**G**). The blot shown is representative of independent biological replicates showing comparable patterns. Actin was used as a loading control (38–49 kDa). Microdensitometric analysis of CDKA band intensity was performed using GelAnalyzer software to compare relative protein abundance across treatments (**H**). The densitometric profiles shown illustrate the distribution of signal intensity within representative bands. Quantitative comparisons between treatments were performed using densitometric values obtained from independent biological replicates (*n* = 3; each representing an independent incubation experiment). Statistical significance: * *p* < 0.05 (one-way ANOVA followed by Tukey’s multiple comparison test).

The level of CDKA kinase was assessed directly in meristematic cells using immunodetection (Figure 4A–E,A′–E′,A″–E″), with quantitative fluorescence intensity analysis (Figure 4F). These results were further validated by Western blotting (Figure 4G), combined with densitometric analysis of band intensities using GelAnalyzer 23.1.1 software (Figure 4H). Both immunofluorescence and Western blot analyses indicated comparable CDKA levels in control seedlings (Figure 4A) and those treated with the emulsifier (Figure 4B), with relative fluorescence intensity of approximately 160 a.u. (Figure 4F) and relative band intensities of 59 and 53 a.u., respectively (Figure 4G,H). Cadmium exposure resulted in a slight decrease in CDKA signal, reflected in both fluorescence intensity (128 a.u.; Figure 4C,F) and band intensity (46 a.u.; Figure 4G,H), indicating a modest reduction of this protein in meristematic cells. Treatment with emulsified TO alone did not significantly alter CDKA levels compared to the control, as confirmed by both immunofluorescence images (Figure 4D,F) and Western blot analysis (54 a.u.; Figure 4G,H). Notably, the presence of TO during cadmium exposure partially prevented this slight decrease, with fluorescence intensity measured at 138 a.u. and band intensity at 52 a.u. (Figure 4E–H).

**Figure 5:**
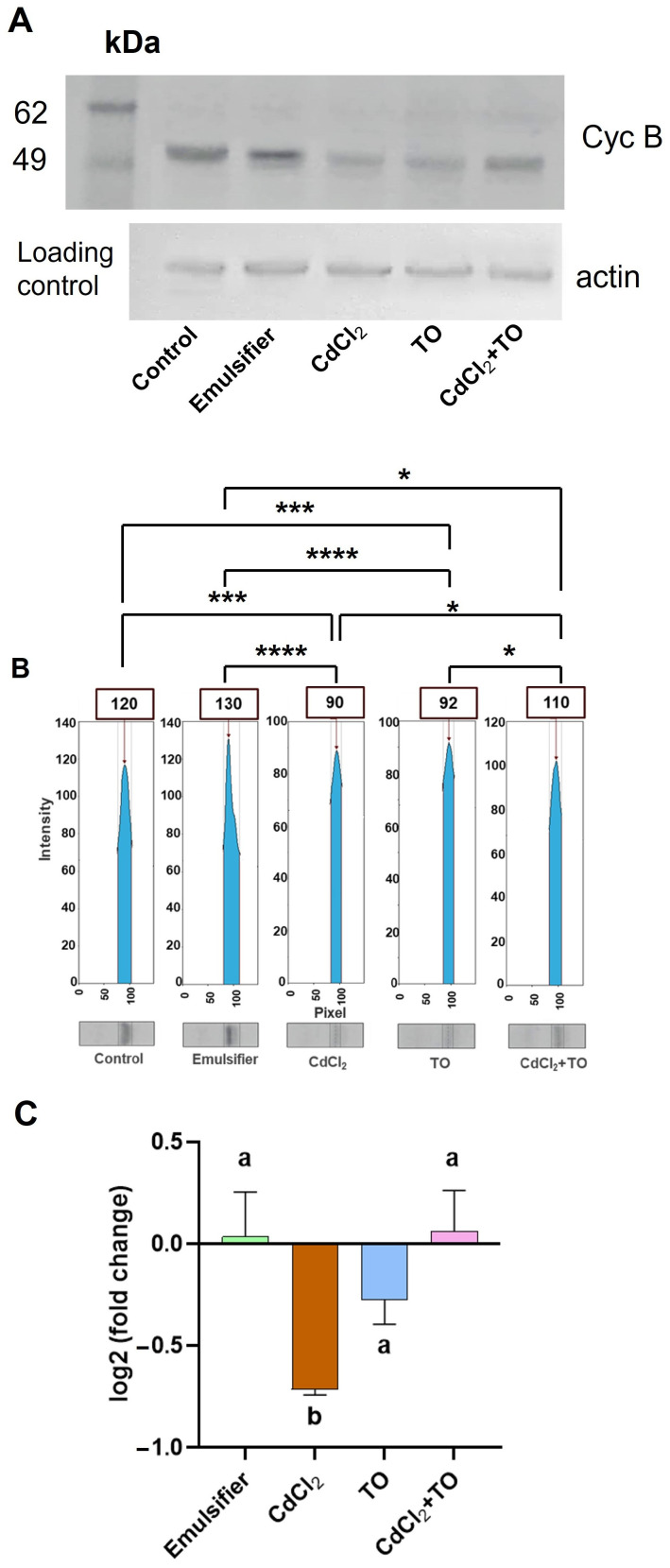
Western blot analysis of CycB in protein extracts obtained from the apical region of *V. faba* primary roots after 24 h of incubation with water—Control, emulsifier, CdCl_2_, thyme essential oil (TO) and a combination of CdCl_2_ and TO (**A**). The blot shown is representative of independent biological replicates showing comparable patterns. Actin was used as a loading control (38–49 kDa). Microdensitometric analysis of CycB band intensity was performed using GelAnalyzer software to compare relative protein abundance across treatments (**B**). The densitometric profiles shown illustrate the distribution of signal intensity within representative bands. Quantitative comparisons between treatments were performed using densitometric values obtained from independent biological replicates *(n* = 3; each representing an independent incubation experiment). Statistical significance: **** *p* < 0.0001, *** *p* < 0.001, * *p* < 0.05 (one-way ANOVA followed by Tukey’s multiple comparison test). Gene expression of *CycB* in the apical region of primary roots was assessed by qPCR and is presented as log_2_ (fold change) relative to the control (**C**). Values are shown as the mean (±SD, *n* = 3 biological replicates; each representing an independent incubation experiment). Statistical significance is indicated by different letters (a, b); means sharing at least one common letter are not significantly different at *p* < 0.05 (one-way ANOVA followed by Tukey’s multiple comparison test).

The level of CycB in meristematic cells was determined by Western blotting, complemented by densitometric analysis of band intensities using GelAnalyzer software (Figure 5A,B). These results were independently verified by qPCR (Figure 5C), enabling quantitative assessment of transcript levels of the *CycB* gene. Both Western blot and qPCR analyses showed comparable CycB protein abundance and transcript levels in control seedlings and those treated with the emulsifier alone (Figure 5A–C). Cadmium exposure led to a clear reduction in CycB protein level, accompanied by decreased transcript abundance, indicating an inhibitory effect of Cd on both protein accumulation and gene expression. Interestingly, despite maintaining mitotic activity comparable to the control in root meristems, treatment with emulsified TO alone also resulted in a marked reduction in CycB protein level and transcript level relative to the control. In contrast, the combined CdCl_2_ + TO treatment maintained CycB protein levels close to control values and was associated with elevated transcript levels, as confirmed by qPCR (Figure 5A–C).

### 2.4. Immunocytochemical Analysis of Cytoskeletal Organization

The pronounced decline in mitotic activity in Cd-exposed cells, accompanied by reduced CycB transcript and protein levels—a major regulator of cell cycle progression—prompted further analysis of microtubule organization, as these structures are responsible for the formation of dynamically reorganized karyokinetic and cytokinetic spindles. Since microtubules are formed by polymerization of α/β-tubulin heterodimers, β-tubulin distribution was examined by immunocytochemistry using fluorescence microscopy.

**Figure 6:**
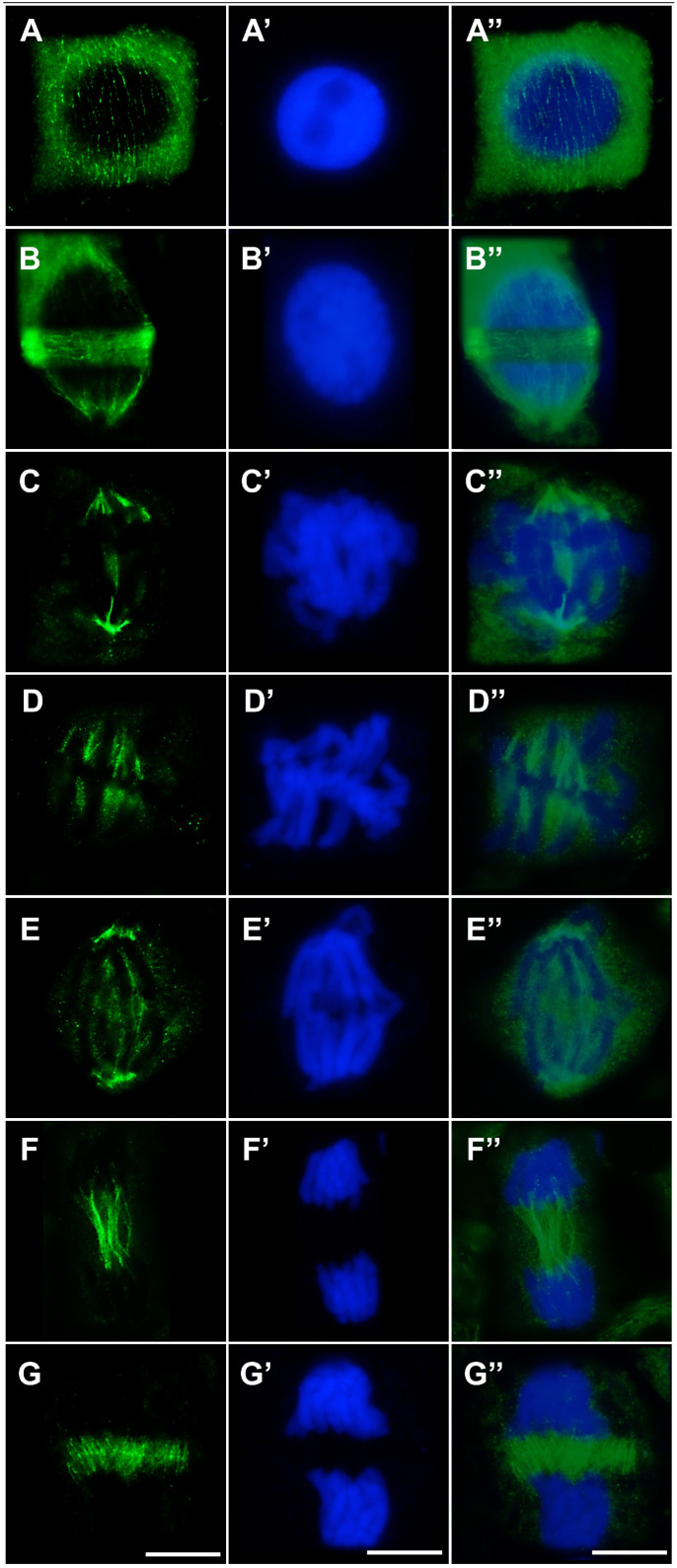
Immunocytochemical detection of β-tubulin (**A**–**G**) in primary root meristem cells of *V. faba* after 24 h of incubation in water—Control. Corresponding images of cell nuclei stained with DAPI (**A′**–**G′**) and merged images (**A″**–**G″**). Scale bar = 10 µm.

**Figure 7:**
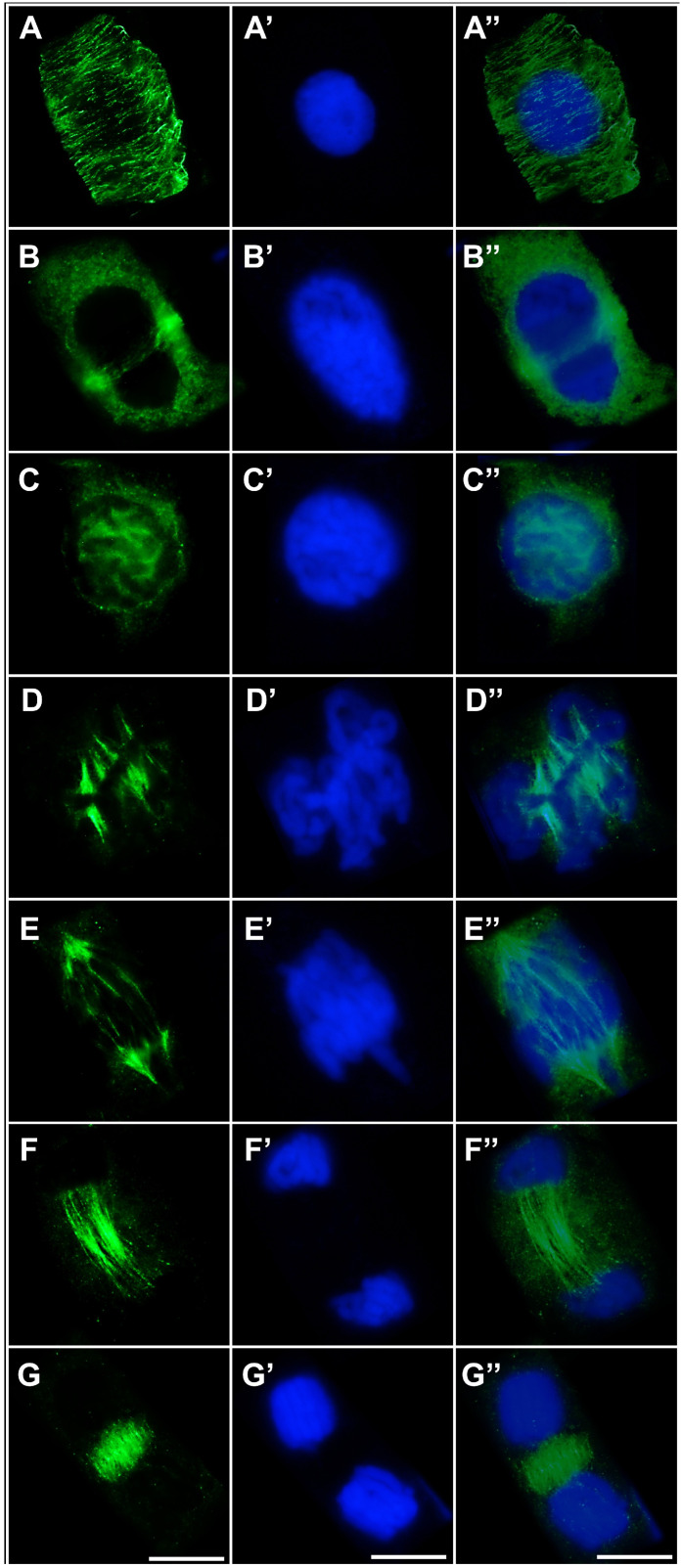
Immunocytochemical detection of β-tubulin (**A**–**G**) in primary root meristem cells of *V. faba* after 24 h of incubation in emulsifier. Corresponding images of cell nuclei stained with DAPI (**A′**–**G′**) and merged images (**A″**–**G″**). Scale bar = 10 µm.

In both control root meristem cells (Figure 6A–G,A′–G′,A″–G″) and cells treated with the emulsifier (Figure 7A–G,A′–G′,A″–G″), microtubule organization at successive stages of the cell cycle appeared normal. During interphase, cortical microtubules formed a regular network of fine, parallel fibers surrounding the cell (Figure 6A–A″ and Figure 7A–A″). In cells preparing for division, these arrays are reorganized into a distinct and continuous preprophase band composed of parallel microtubules encircling the nucleus at the equatorial plane and marking the future division site (Figure 6B–B″ and Figure 7B–B″). In prophase and prometaphase, further reorganization of the preprophase band gave rise to a dense microtubule array concentrated around the disassembling nuclear envelope (Figure 6C–C″ and Figure 7C–C″). At metaphase, prominent kinetochore microtubules formed a typical bipolar spindle (Figure 6D–D″ and Figure 7D–D″). During early anaphase, kinetochore microtubules responsible for chromatid segregation were accompanied by polar microtubule bundles (Figure 6E–E″ and Figure 7E–E″). These bundles became particularly conspicuous in late anaphase, contributing to the formation of the midzone required for subsequent cell plate assembly (Figure 6F–F″ and Figure 7F–F″). In late telophase, following the disassembly of the karyokinetic spindle, a well-developed phragmoplast was observed within this region (Figure 6G–G″ and Figure 7G–G″).

**Figure 8:**
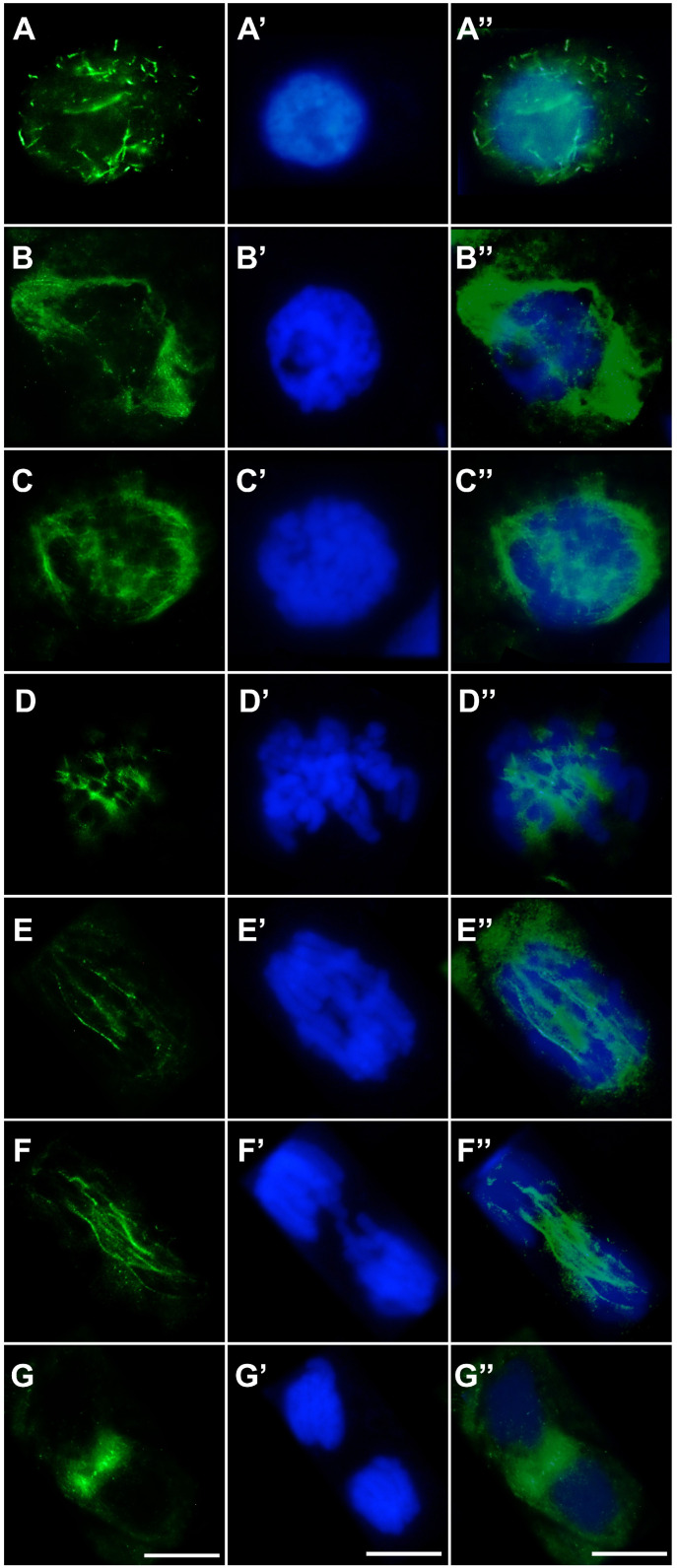
Immunocytochemical detection of β-tubulin (**A**–**G**) in primary root meristem cells of *V. faba* after 24 h of incubation in CdCl_2_. Corresponding images of cell nuclei stained with DAPI (**A′**–**G′**) and merged images (**A″**–**G″**). Scale bar = 10 µm.

In cells exposed to cadmium, pronounced disturbances in microtubule organization and their reorganization into mitotic spindle structures were observed (Figure 8A–G,A′–G′,A″–G″). Already at interphase, cortical microtubules failed to form the regular, parallel arrays characteristic of control cells. Instead, short and frequently thickened rod-like microtubules, typical of cadmium stress, predominated and were chaotically distributed throughout the cytoplasm (Figure 8A–A″). Cd also markedly affected the organization of the preprophase band, which was often incomplete or discontinuous and showed a loss of the typical parallel microtubule alignment (Figure 8B–B″). In prophase, microtubules derived from preprophase band reorganization did not establish a well-ordered fibrous array concentrated around the nuclear envelope; rather, they appeared as a dispersed mass of short, entangled structures (Figure 8C–C″). At metaphase, the mitotic spindle was clearly deformed, and kinetochore microtubule bundles were shorter and less abundant than in control cells (Figure 8D–D″). During early anaphase, kinetochore fibers that normally formed distinct bundles toward the spindle poles displayed a dispersed pattern, while polar microtubules in the central spindle region were poorly organized, irregularly arranged, and frequently fragmented (Figure 8E–E″). In telophase, marked disturbances were observed in the organization of polar microtubules preceding phragmoplast formation. Instead of the uniform, parallel bundles typical of control cells, microtubules formed heterogeneous structures differing in thickness and apparent polymerization status (Figure 8F–F″). In late telophase, the phragmoplast showed pronounced deviations from normal architecture: rather than evenly distributed, dense, and parallel microtubules of comparable length, short, intensely fluorescent, thickened bundles were observed, often lacking coherent orientation and embedded within regions of weak and irregular fluorescence signal (Figure 8G–G″).

**Figure 9:**
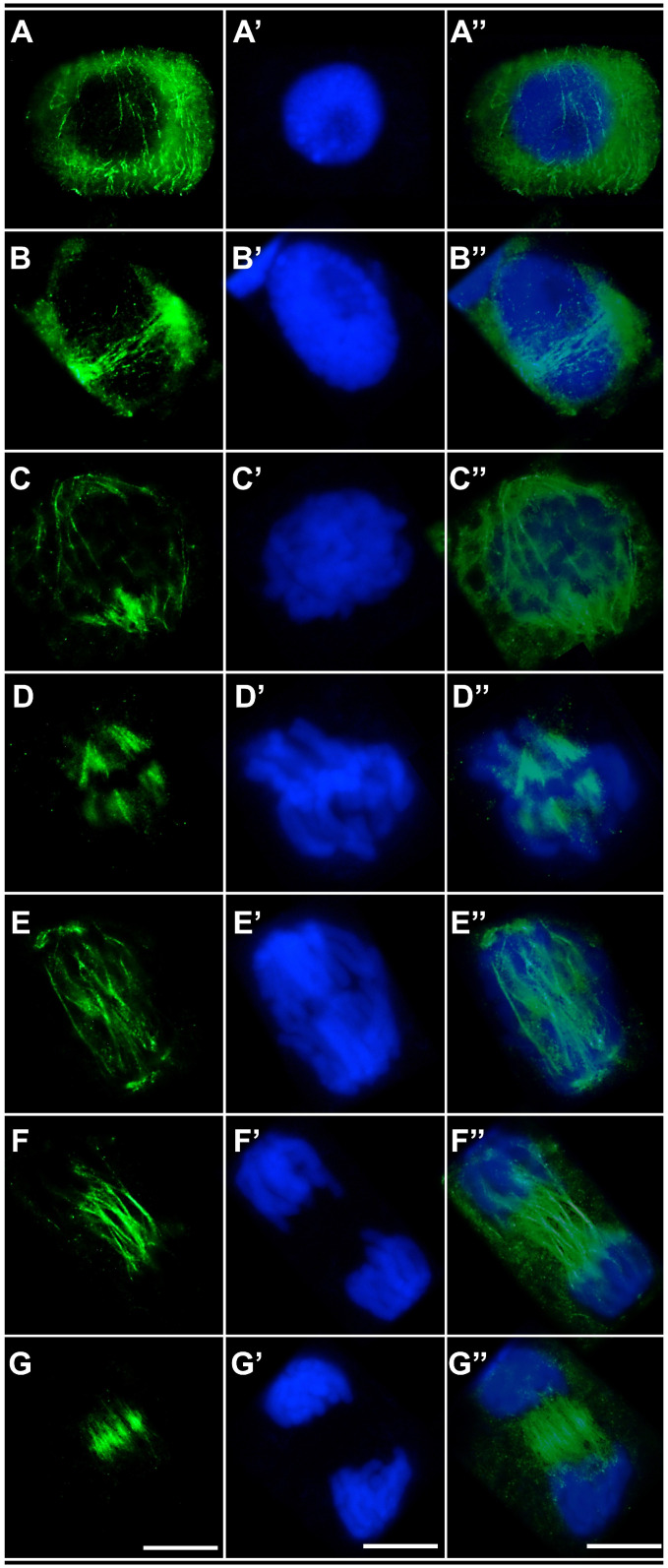
Immunocytochemical detection of β-tubulin (**A**–**G**) in root meristem cells of *V. faba* after 24 h of incubation in thyme essential oil—TO. Corresponding images of cell nuclei stained with DAPI (**A′**–**G′**) and merged images (**A″**–**G″**). Scale bar = 10 µm.

In cells exposed to the emulsified form of TO, microtubule organization did not deviate from the pattern observed in control cells (Figure 9A–G,A′–G′,A″–G″). Both cortical microtubules and preprophase band microtubules in interphase (Figure 9A–A″,B–B″), as well as elements of the mitotic spindle in prophase (Figure 9C–C″), metaphase (Figure 9D–D″), early and late anaphase (Figure 9E–E″,F–F″), and telophase (Figure 9G–G″), exhibited a well-ordered structure typical for each cell cycle stage.

**Figure 10:**
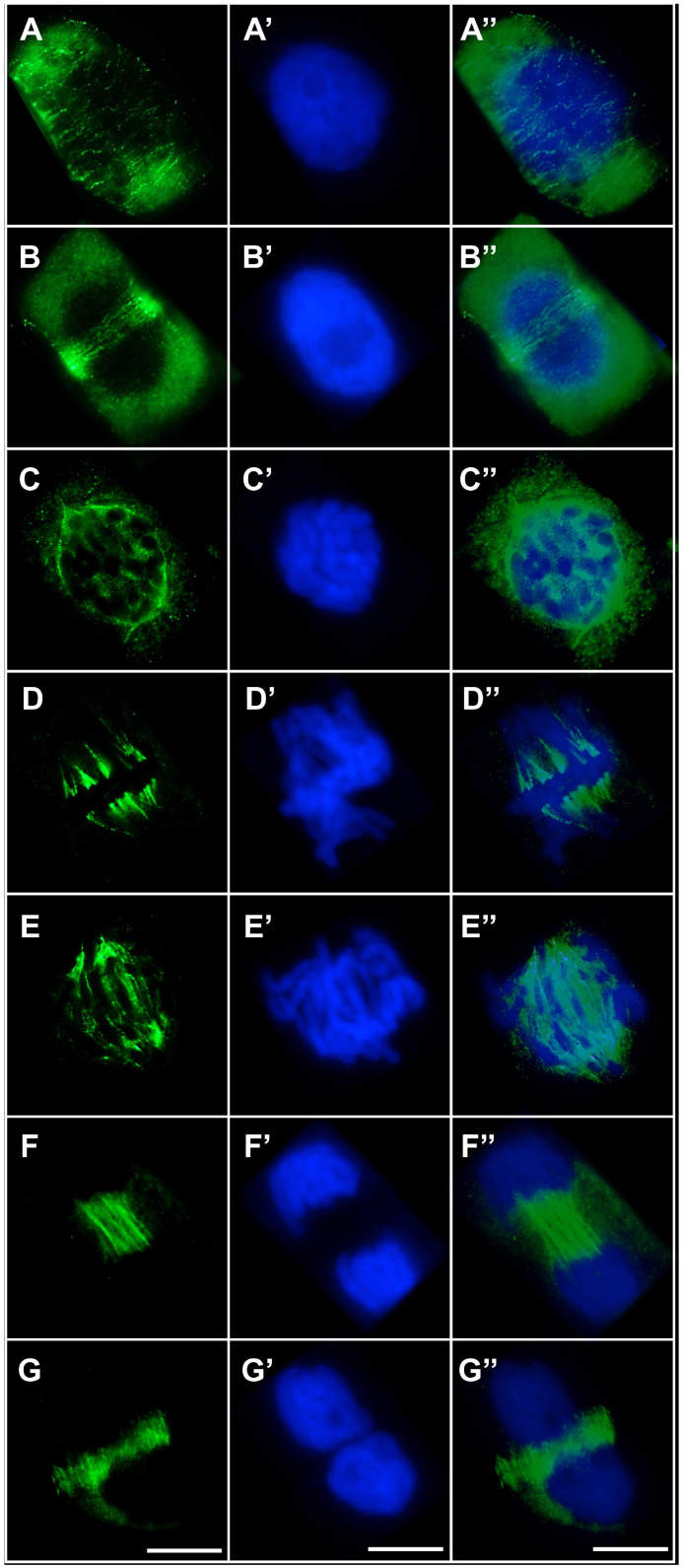
Immunocytochemical detection of β-tubulin (**A**–**G**) in primary root meristem cells of *V. faba* after 24 h of incubation in combination of CdCl_2_ and thyme essential oil—TO. Corresponding images of cell nuclei stained with DAPI (**A′**–**G′**) and merged images (**A″**–**G″**). Scale bar = 10 µm.

A similar pattern was observed in cells treated simultaneously with CdCl_2_ and TO (Figure 10A–G,A′–G′,A″–G″). Cortical microtubules maintained a regular arrangement of long, parallel fibers; the mitotic spindle displayed normal bipolar symmetry with properly organized kinetochore and polar microtubule bundles, and the phragmoplast formed a regular structure composed of short, parallel-aligned microtubules.

In light of the observed disturbances in microtubule structure and organization in Cd-exposed cells, we also analyzed the remodeling of actin filaments, which constitute an essential component of the cytoskeleton in dividing cells. Actin filaments, supporting microtubule function, undergo dynamic rearrangements, forming both locally organized, dense bundles and thin, elongated filaments that contribute to a more extensive network.

**Figure 11:**
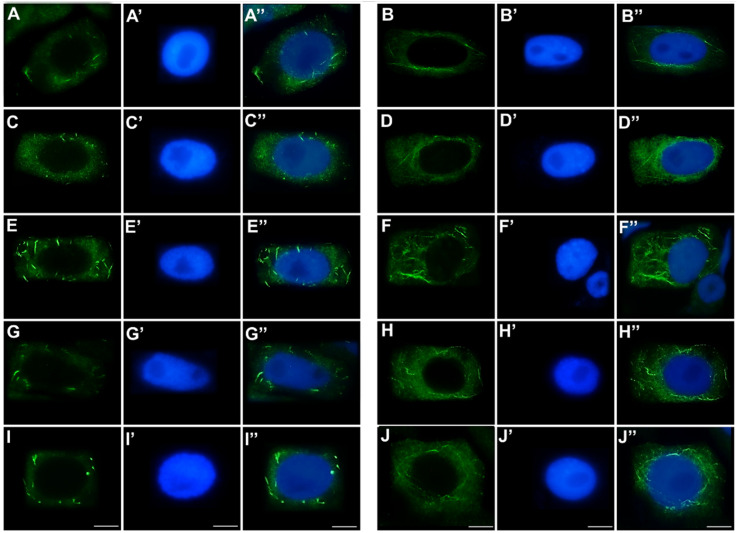
Immunocytochemical detection of actin in primary root meristem cells of *V. faba* after 24 h of incubation in water—Control (**A**,**B**), emulsifier (**C**,**D**), CdCl_2_ (**E**,**F**), thyme essential oil—TO (**G**,**H**), and a combination of CdCl_2_ and TO (**I**,**J**). Corresponding images of cell nuclei stained with DAPI (**A′**–**J′**) and merged images (**A″**–**J″**). Scale bar = 10 µm.

In control meristem cells and those treated with the emulsifier, both filament forms were present: compact, intensely fluorescent bundles of short filaments (Figure 11A,C), as well as a fine, diffuse network composed of long filaments extending along the longitudinal axis of the cells (Figure 11B,D). Following 24 h of CdCl_2_ exposure, the overall filament arrangement remained comparable to the control, but both the short bundles and the longer filaments exhibited noticeable thickening and increased fluorescence intensity, suggesting additional lateral filament interactions (Figure 11E,F). In cells treated with the emulsified form of TO, the organization, packing, thickness, and orientation of filaments resembled those observed in control cells (Figure 11G,H). In the case of simultaneous exposure to CdCl_2_ and TO, only moderate thickening of short actin bundles was observed, while the longer filaments retained a control-like appearance, with no obvious increase in fluorescence intensity or disruption of the filament network (Figure 11I,J).

### 2.5. Immunocytochemical Analysis of Mitotic Epigenetic Modifications of Histone H3

**Figure 12:**
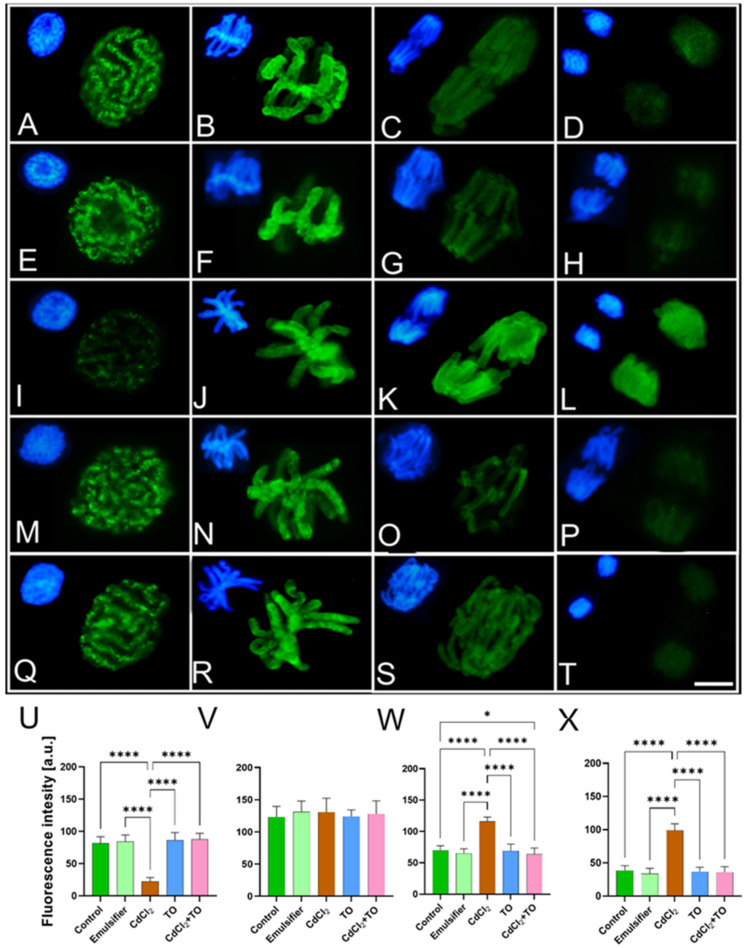
Immunofluorescence detection of H3T3Ph in mitotic figures of the primary root meristem cells of *V. faba* after 24 h of incubation in water—Control (**A**–**D**) emulsifier (**E**–**H**), CdCl_2_ (**I**–**L**), thyme essential oil (TO) (**M**–**P**), and a combination of CdCl_2_ and TO (**Q**–**T**). Scale bar = 10 μm. Corresponding images of cell nuclei stained with DAPI, embedded in the upper left corners of each image. Mean (± SD; *n* = 3 biological replicates; each representing an individual seedling) intensity of H3T3Ph labeling in prophase (**U**), metaphase (**V**), anaphase (**W**) and telophase (**X**); approximately 30 nuclei were analyzed per treatment. Statistical significance: **** *p* < 0.0001, * *p* < 0.05 (one-way ANOVA followed by Tukey’s multiple comparison test).

**Figure 13:**
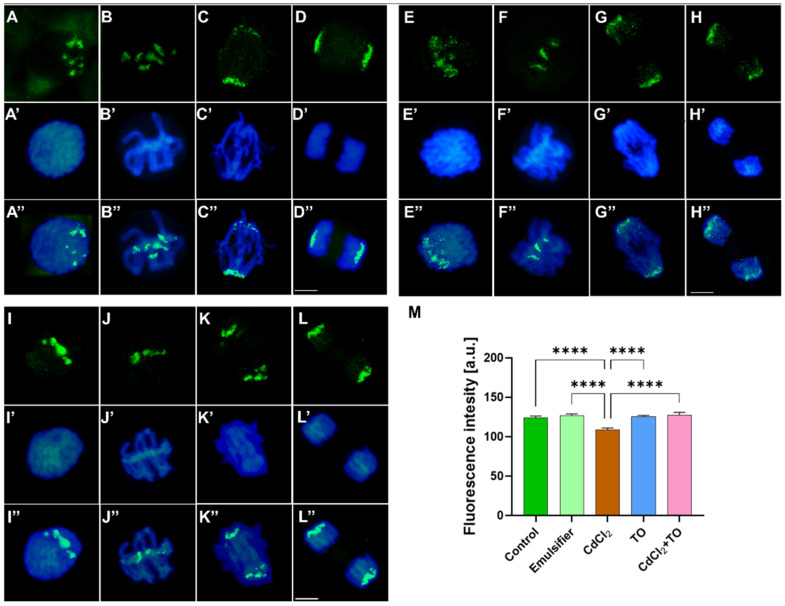
Immunofluorescence detection of H3S10Ph in mitotic figures of the primary root meristem cells of *V. faba* after 24 h of incubation in water—Control (**A**–**D**), CdCl_2_ (**E**–**H**), and a combination of CdCl_2_ and thyme essential oil—TO (**I**–**L**). Corresponding images of cell nuclei stained with DAPI (**A’**–**L’**) and merged images (**A″**–**L″**). Images for the emulsifier and TO were omitted since they showed no differences compared with the control. Scale bar = 10 µm. Mean (± SD; *n* = 5 biological replicates; each representing an individual seedling) intensity of H3S10Ph labeling in mitotic figures; approximately 50 nuclei were analyzed per treatment (**M**). Statistical significance: **** *p* < 0.0001 (one-way ANOVA followed by Tukey’s multiple comparison test).

Given that both chromatin condensation into mitotic chromosomes and the proper function of the cytoskeleton required for chromatid segregation and cell division depend on the recruitment of specific mitotic regulators—which in turn are influenced by dynamic epigenetic modifications—we analyzed selected, commonly studied histone H3 modifications. Specifically, phosphorylation of threonine 3 (H3T3Ph; Figure 12) and serine 10 (H3S10Ph; Figure 13) was examined using immunocytochemical methods.

In the analysis of H3T3Ph, meristem cells from control seedlings and those treated with the emulsifier exhibited a strong immunopositive signal during prophase, appearing as numerous distinct foci of variable size distributed along the arms of condensing chromosomes (Figure 12A,E,U). During metaphase, the entire chromosomes displayed intense mosaic fluorescence, with the strongest labeling localized in the pericentromeric regions (Figure 12B,F,V). Following sister chromatid separation in anaphase, the H3T3Ph signal was markedly reduced, and chromosomes in telophase were devoid of specific labeling (Figure 12C,D,G,H,W,X).

Cells exposed to CdCl_2_ showed clear alterations in the pattern of this epigenetic modification (Figure 12I–L,U–W,X). Compared with control, the H3T3Ph signal in prophase, while retaining its focal pattern along condensing chromosomes, was noticeably weaker (Figure 12I,U). In metaphase, the intensity and labeling pattern were not significantly different from those observed in control conditions (Figure 12J,V). However, in subsequent mitotic stages (anaphase and telophase), the H3T3Ph signal did not undergo the typical attenuation but remained at a relatively high level, similar to that in metaphase (Figure 12K,L,W,X), indicating a disruption of the normal phase-dependent dynamics of histone H3 phosphorylation at threonine 3.

In cells treated with emulsified TO, both the labeling pattern and fluorescence intensity of H3T3Ph were comparable to those observed in control cells (Figure 12M–P,U–W,X), suggesting preserved physiological dynamics of this modification across mitotic stages. In cells simultaneously exposed to CdCl_2_ and TO, the first two stages of mitosis exhibited a H3T3Ph signal pattern and intensity similar to the control (Figure 12Q,R,U,V). The main difference was the prolonged presence of a very weak signal extending into anaphase, during which H3T3Ph phosphorylation remained detectable along the arms of separating sister chromatids (Figure 12S,W). By telophase, the labeling pattern normalized and resembled that of control cells (Figure 12T,X).

In the analysis of H3S10Ph, meristem cells from control seedlings (Figure 13A–D) and those treated with the emulsifier exhibited an identical labeling pattern. The H3S10Ph signal appeared already in early prophase as intensely fluorescent clusters localized in the strongly condensed pericentromeric regions of chromosomes, concentrated near one pole of the nucleus (Figure 13A–A″). This arrangement reflects pronounced chromosome polarization, consistent with Rabl’s model, which proposes an ordered, non-random organization of chromosomes in the interphase nucleus. During metaphase, the H3S10Ph signal was clearly visible in the pericentromeric regions of chromosomes aligned at the metaphase plate (Figure 13B–B″). During anaphase, the labeled regions migrated with the centromeres of separating sister chromatids toward opposite poles of the cell (Figure 13C–C″). In telophase, the H3S10Ph signal remained confined to narrow, brightly fluorescent regions corresponding to the centromeres of single-chromatid daughter chromosomes that had reached the cell poles (Figure 13D–D″).

In cells incubated with CdCl_2_, the overall localization of the H3S10Ph signal did not markedly differ from that observed in control cells (Figure 13E–H,E′–H′,E″–H″), remaining concentrated in the pericentromeric regions throughout mitosis. However, subtle changes in signal morphology and dispersion were evident. In prophase, fluorescent foci appeared more dispersed and finely granular (“sandy”) compared with the distinct clustered structures seen in control cells (Figure 13E–E″). During metaphase, the foci were less numerous (Figure 13F–F″). In anaphase and telophase, a weak H3S10Ph signal persisted not only in the pericentromeric regions but also along the chromosome arms (Figure 13G–G″,H–H″), which was not observed in control cells. Quantitative fluorescence analysis revealed a reduction in H3S10Ph signal intensity in CdCl_2_-treated meristems compared with the other experimental variants (Figure 13M), indicating that cadmium affects histone H3 phosphorylation at serine 10, despite maintaining the relative localization of the signal. In cells treated with TO and in cells simultaneously exposed to CdCl_2_ and TO (Figure 13I–L,I′–L′,I″–L″), the H3S10Ph labeling pattern closely resembled that of control cells in terms of signal localization, dynamic changes throughout mitotic stages, and fluorescence intensity (Figure 13M).

## 3. Discussion

Mitosis is a tightly regulated, multi-level process that requires precise coordination of cell cycle regulators (CDK–Cyc complexes), establishment of mitosis-specific histone modifications, preparation of chromatin for condensation, and dynamic reorganization of the microtubule–actin cytoskeleton [66,67,68]. Disruption of any of these components can result in delays, abnormal progression, or complete inhibition of mitosis. Cadmium, described both in our previous studies [59,60] and in the broader literature as a potent genotoxic and pro-oxidative agent, significantly interferes with key mechanisms regulating cell cycle progression [69,70]. The present data further suggest that TO, known for its antioxidant properties [59,71], has the potential to mitigate cadmium-induced mitotic disturbances.

### 3.1. Cadmium Effects on Meristematic Cell Division and Root Growth in V. faba Seedlings

The meristematic tissue responsible for primary root growth is particularly sensitive to abiotic stresses, including cadmium toxicity [72]. In the present study, cadmium exposure reduced root elongation by approximately 50% and decreased the mitotic index nearly tenfold, confirming strong inhibition of organ growth through impaired cell proliferation [61,73,74]. Reduced mitotic activity may result from cell cycle arrest during interphase, inhibition of mitotic entry, or disturbances in mitotic progression, as suggested by the accumulation of prophase cells and the reduced proportion of anaphase and telophase figures.

Studies on *Zea mays* meristematic tissue have shown that cadmium accumulation prolongs the cell cycle, partly by inhibiting the G1-to-S phase transition [9]. A similar mechanism was indicated by our previous observations in *V. faba*, where cadmium reduced the number of replicating cells and caused their accumulation in early S phase, suggesting slowed DNA replication [59]. As a strong inducer of oxidative stress, cadmium can also cause DNA damage, that activates the DNA damage response (DDR), which delays cell cycle progression until lesions are repaired [75,76]. Our studies in *V. faba* confirmed the genotoxic effects of cadmium [59], indicating that replication stress likely contributed to the reduced mitotic activity observed here.

Cadmium-treated roots also exhibited increased chromatin condensation in interphase nuclei, visible as compact and irregular regions of genetic material, as observed in the present study. These structural changes may result from cadmium-induced epigenetic modifications, including DNA methylation and histone modifications, associated with chromatin compaction and transcriptional repression, as demonstrated in our parallel study [60]. These alterations may prolong interphase and further reduce the proportion of dividing cells. However, disturbances in the regulation of the G2/M transition in cells that have completed interphase cannot be excluded. Therefore, the activity of key regulators of this transition, CDKA and CycB, was analyzed.

### 3.2. Cadmium Disrupts Cell Cycle Regulators: CDKA and CycB

Cell cycle progression depends on the activity of CDK–Cyc complexes, in which relatively stable CDKs are regulated by periodic cyclin accumulation, enabling entry into mitosis [22]. Disturbances in the levels of these components can arrest the cell cycle [77] and may occur under environmental stress, DNA damage, or exposure to toxic agents [78,79,80,81,82].

**Figure 14:**
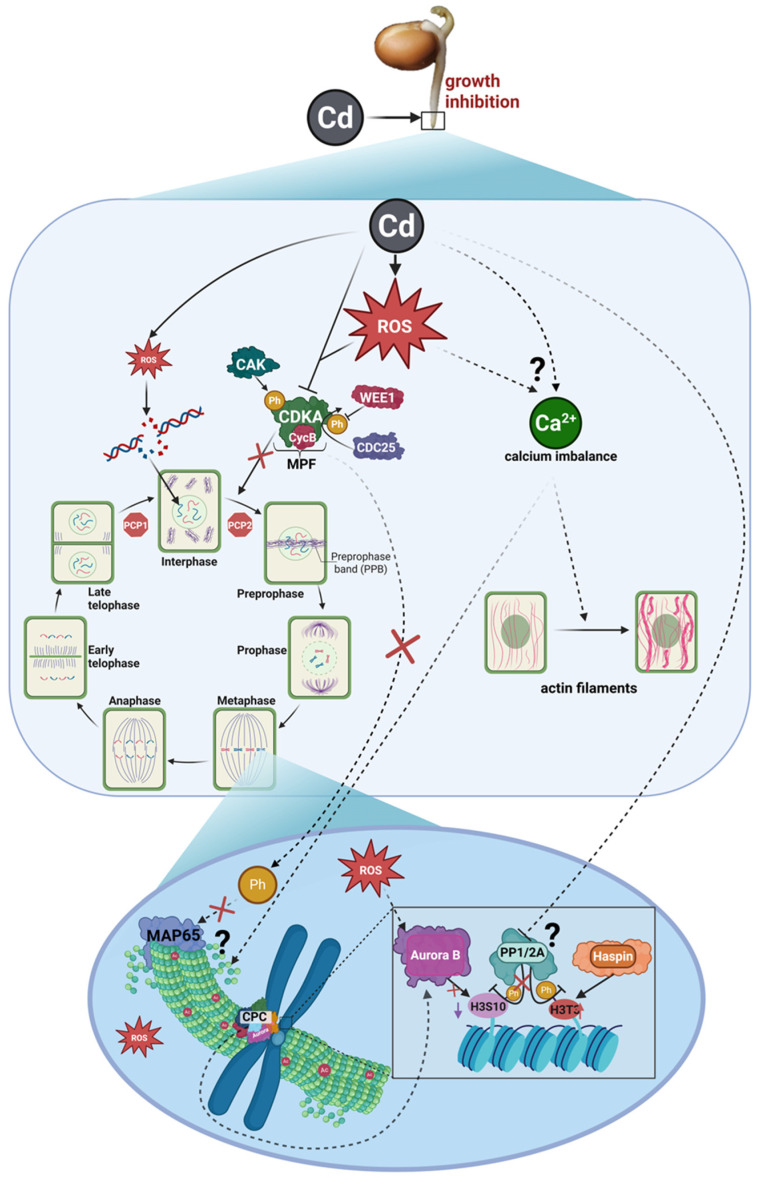
Proposed mechanism of cadmium (Cd) effects on mitotic progression. Arrows indicate the direction of processes or signaling pathways; arrows with pointed tips denote activation or enhancement, whereas arrows with blunt (flat) tips denote inhibition or suppression. Solid arrows indicate relationships directly supported by experimental data obtained in our study. Dashed arrows and question marks indicate proposed mechanisms inferred from prior literature and not directly tested here. Cross symbols (X) indicate inhibition or suppression of the indicated processes or interactions. Cd enters the cell and induces overproduction of reactive oxygen species (ROS). Elevated ROS levels are proposed to disrupt calcium metabolism and affect the formation or activity of CDK–Cyc complexes, resulting in cell cycle arrest at the G2/M transition and reduced mitotic entry. Reduced levels/activity of the M-phase Promoting Factor (MPF) may impair inhibitory phosphorylation of MAP65. Consequently, MAP65 may contribute to increased microtubule stabilization, which could affect proper spindle organization. Additionally, Cd-induced stress may interfere with protein phosphatases PP1 and PP2A, thereby potentially altering the regulation of mitotic kinases such as Aurora B and Haspin. Disruption of histone H3 phosphorylation dynamics may in turn affect chromatin organization. Collectively, these proposed alterations could contribute to reduced mitotic index and impaired root growth. Created in BioRender. Gocek-Szczurtek N. (2026). https://BioRender.com/on5o09l.

Exposure to CdCl_2_ caused a slight but distinct reduction in the conserved CDKA protein level. Literature data indicate that cadmium effects on cell cycle regulators are dose-dependent. Moderate concentrations can increase the expression of genes such as *CDKA1* or the cell cycle inhibitor *WEE1* [8], or may not cause significant changes in *CDKA* levels, as reported in *Glycine max* cultures [83]. In contrast, severe toxic stress suppresses the expression of cell cycle-related genes [8]. The relatively high concentration used in this study (175 µM CdCl_2_) therefore likely reflects stress conditions sufficient to disrupt even relatively stable components of the cell-cycle regulatory machinery. The modest magnitude of this change may reflect homeostatic control of CDKA levels, although it does not eliminate its sensitivity to stress. Similar trends have been reported in *A. thaliana* meristems exposed to salt stress, where NaCl-induced reductions in *CDC2aAt* and *CDC2bAt* expression correlated with decreased mitotic activity [84]. Although salt and cadmium stress differ in their primary triggers, both induce secondary oxidative stress, leading to ROS overproduction, redox imbalance, DNA damage, and activation of cell cycle checkpoints [59,75,84,85]. Under such conditions, reduced CDKA activity can contribute to cell cycle arrest and prevent entry into mitosis in cells with damaged genomes (Figure 14). Cadmium stress may also induce broader epigenetic and transcriptional reprogramming [60,86,87]. In this context, the observed decrease in CDKA could partly reflect general repression of proliferation-associated genes under severe stress rather than specific targeting of this regulator. In contrast to the moderate reduction in CDKA, a pronounced decrease in CycB protein levels and transcript abundance was observed after 24 h exposure to 175 µM CdCl_2_. Similar repression of B-type cyclins under cadmium stress has been reported in *A. thaliana* root cells [86,88], *Z. mays* meristems and leaves [9], and *G. max* suspension cultures [83]. These findings suggest that inhibition of B-type cyclin expression represents a key mechanism limiting mitotic activity and preventing cells that have completed interphase from entering mitosis (Figure 14).

### 3.3. Cadmium-Induced Alterations in Cytoskeleton: Actin Filaments and Microtubules

Cadmium, even at low concentrations (0.25 µM), is known to disrupt cytoskeletal organization, affecting both microtubules and actin filaments and thereby impairing cell cycle progression and root growth [61,73,89,90,91]. In the present study, exposure of *V. faba* seedlings to 175 µM CdCl_2_ caused pronounced disturbances in the microtubule-actin cytoskeleton of root meristems. In interphase cells, the cortical microtubule network lost its regular orientation and appeared as short, rod-like structures. Similar alterations have been reported in the green alga *Spirogyra decimina* [92] and in higher plant cells, where microtubules also fragmented into segments of variable lengths [63,90,93]. Disruption of cortical microtubules impaired preprophase band formation and the subsequent assembly of a functional mitotic spindle, potentially contributing to delays in early mitotic stages and to the increased proportion of prophase cells observed in this study. Comparable effects have been reported in cadmium-treated root cells of *Salix matsudana*, where spindle fibers formed condensed arrays in metaphase and aggregated during anaphase and telophase [93] as we also observed. Microtubule aggregation, which reduces their dynamic behavior, likely contributed to the lower frequency of anaphase and telophase cells, prolonging mitosis and limiting primary root growth. Similar effects on spindle stability and mitotic stage distribution have been reported under cadmium stress [61].

Microtubule aggregation and difficulties in their reorganization may reflect adaptive cellular responses to oxidative stress and disturbed redox homeostasis under cadmium exposure [75]. These responses may involve ROS-induced post-translational modifications of tubulin, such as acetylation and detyrosination, which have been shown to increase microtubule mechanical resistance but reduce their dynamic remodeling [94]. Similar stress-induced stabilization has been reported in sponge cells (*Clathrina clathrus*) [95] and in *G. max* root cells exposed to cadmium [90]. However, excessive stabilization ultimately impairs microtubule function [94].

The regulation of microtubule organization also involves microtubule-associated proteins (MAPs) [36], including MAP65-1, which bundles parallel microtubules [96,97,98]. Literature data indicate that it can mediate atypical tubulin polymers under ROS disturbances [99] and preferentially binds stabilized filaments, enhancing local microtubule density [100]. MAP65 activity is controlled by CDKA–CycB dependent phosphorylation: when phosphorylated, it detaches from microtubules, increasing their dynamics [97]. Under cadmium stress, the reduced CDKA and CycB levels observed in our study may therefore affect MAP65 phosphorylation status and potentially favor its association with microtubules. Together with tubulin acetylation and detyrosination, which are reported on in [94], this may contribute to increased bundling and altered organization of microtubules observed in our immunocytochemical analyses.

Cadmium is also known to disrupt Ca^2+^ homeostasis through interactions with membrane channels and calcium-binding proteins. Literature reports indicate that both ROS-mediated pathways and the direct action of CdCl_2_ increase cytosolic Ca^2+^ levels [101,102], and that disturbances in calcium balance can affect microtubule organization and stability [103]. Elevated cytosolic Ca^2+^ promotes depolymerization at microtubule ends, thereby destabilizing these structures [104]. Calcium imbalance may influence not only microtubules but also actin filaments. In our study, cadmium markedly altered the organization of actin filaments in *V. faba* meristematic cells. Thickening of both short bundles and long filaments, reflected by increased fluorescence intensity, suggests enhanced filament aggregation and the formation of less ordered actin structures. Similar effects may result from direct interactions between Cd^2+^ ions and actin, leading to aggregate formation [105]. However, an indirect mechanism related to disturbed calcium signaling cannot be excluded. According to literature reports, elevated cytosolic Ca^2+^ activates signaling cascades that regulate numerous actin-binding proteins involved in controlling filament organization, including processes such as severing, stabilization, or inhibition of elongation [106]. The filament bundling and local stabilization observed in our study may therefore reflect the combined effects of direct Cd^2+^ action and indirect consequences of oxidative stress and disrupted calcium signaling.

The cytoskeletal response to cadmium stress therefore appears to be multilayered and the result of the interplay of several regulatory mechanisms. Changes in tubulin post-translational modifications, modulation of microtubule-associated protein activity, and disturbances in calcium and redox signaling together form an integrated response network in which filament stabilization may represent an adaptive reaction to unfavorable conditions. However, such stabilization, while increasing the mechanical resistance of cytoskeletal structures, may also be associated with reduced flexibility of their organization, as observed in *V. faba* primary root meristems (Figure 14). Such alterations in microtubule organization could therefore contribute to difficulties in chromatid separation and proper segregation of genetic material into daughter nuclei. Future studies using transmission electron microscopy (TEM) will enable detailed ultrastructural analyses of microtubule organization and provide a quantitative framework to complement the qualitative observations presented in this study.

### 3.4. Cadmium-Induced Changes in Mitosis-Related H3 Phosphorylation (H3T3 and H3S10) Patterns

Enhanced chromosome condensation and the presence of “sticky” telomeric regions of sister chromatids—particularly evident in metaphase and promoting their adhesion in anaphase—may result from cadmium–induced mutations in genes encoding non–histone chromatin–associated proteins, as well as from direct interactions of cadmium with histones. Such effects can disrupt chromosome condensation and segregation, manifesting as chromosomal stickiness [107,108]. However, the chromosome abnormalities and spindle defects observed in our study—such as microtubule aggregation and the increased proportion of cells arrested in prophase and metaphase—may also reflect disturbed regulation of mitosis-specific epigenetic histone modifications. In particular, histone H3 phosphorylation forms a functional cascade in which phosphorylation at threonine 3 (H3T3Ph) precedes and facilitates phosphorylation at serine 10 (H3S10Ph). Phosphorylation of H3T3, essential for proper chromosome condensation and spindle assembly checkpoint function, is catalyzed by the AtHaspin kinase identified in *A. thaliana* [109]. This modification acts as a recruitment signal for the chromosomal passenger complex (CPC), a mitosis-specific complex responsible for correcting improper kinetochore–microtubule attachments, ensuring accurate chromosome segregation, and contributing to spindle disassembly. In plants, the CPC includes Aurora B kinases of the AtAUR family (identified in *A. thaliana*), together with homologs of other CPC components known from animal cells [50,110]. Recruitment of the CPC to chromosomes leads to local activation of Aurora B kinases, which in plants phosphorylate H3S10 mainly in pericentromeric regions, a modification associated with maintenance of sister chromatid cohesion and their faithful segregation [51,52].

According to literature, H3T3Ph and H3S10Ph levels are tightly regulated not only by kinases but also by the protein phosphatases PP1 and PP2A [111,112]. Temporal suppression of these enzymes is essential for mitotic entry, while timely reactivation ensures proper progression through mitosis, coordinating mitotic kinases (Haspin and Aurora B), chromatin condensation, microtubule organization, and the metaphase-to-anaphase transition [113,114,115]. PP1 dephosphorylates H3T3, controlling CPC recruitment and Aurra kinase activity, which ultimately affects H3S10 phosphorylation and sister chromatid segregation [116,117]. PP2A regulates phosphorylated centromeric and kinetochore proteins, indirectly modulating CPC and Aurora function in pericentromeric regions [118]. In our study, the H3T3Ph signal, which normally declined during anaphase in control cells, remained elevated in CdCl_2_-treated cells throughout anaphase and telophase. This suggests that cadmium may inhibit PP1/PP2A activity, sustaining Haspin activation and preventing full H3T3 dephosphorylation [119]. Similar mitotic defects, including sticky chromatid ends, have been reported following PP1/2A inhibition with okadaic acid in *V. faba* [114]. Moreover, high Cd concentrations reduce PP1/PP2A gene expression in plants [120,121], and in animal models, cadmium via ROS overproduction also inhibits PP2A [122]. Insufficient phosphatase activity may thus sustain H3T3Ph impair CPC/Aurora recruitment, limit H3S10 phosphorylation, and delay metaphase-to-anaphase progression, as observed in our experiments. Reduced H3S10Ph could also result from altered chromatin architecture limiting the accessibility of the S10 residue or from impaired activation of Aurora kinases by CDK–Cyc complexes [123], consistent with our observations of decreased CycB and modestly reduced CDKA. Literature further indicates that ROS can mislocalize Aurora B along chromosome arms without changing its total levels [124,125]. In line with this, we observed subtle spreading of the H3S10Ph along chromosome arms, suggesting a shift in Aurora B localization away from centromeres and related mitotic dysfunction (Figure 14).

### 3.5. Thyme Oil Mitigates Cadmium-Induced Disruptions in Mitosis and Its Regulatory Mechanisms

A promising strategy to enhance plant tolerance to cadmium, is the use of natural compounds with protective properties. Since cadmium is a strong inducer of oxidative stress [75,126] and TO has well-documented antioxidant activity [127,128,129], TO was tested here as a potential protective agent. Applied alone at 0.03% (*v*/*v*), TO had no effect on primary root growth, mitotic progression, or mitosis-related structural changes in *V. faba*. The apparent disparity between the reduced CycB transcript and protein levels and the unchanged mitotic activity in the TO-treated series can likely be explained by the regulatory characteristics of the G2/M transition. Although TO alone caused a decrease in CycB abundance, the magnitude of this reduction may have remained within the tolerance range of the mitotic regulatory system and therefore did not translate into a measurable decrease in the mitotic index. This interpretation is consistent with the hysteresis behavior of the G2/M transition, in which mitotic entry depends on threshold activation of CDK–Cyc complexes rather than on linear changes in cyclin abundance [130]. Importantly, the decrease in CycB levels was not accompanied by changes in CDKA protein abundance. This observation is consistent with the established regulatory framework of the plant cell cycle, where CDK proteins are typically maintained at relatively stable levels, whereas cyclins represent the more dynamically regulated component controlling CDK activity [131,132,133]. The modest CycB reduction may result from redox-dependent regulation of cell-cycle genes. ROS act not only as damaging agents but also as signaling molecules controlling proliferation-related transcription [134]. TO’s antioxidant activity, including enhanced SOD, CAT, and APX activities reported in the literature [55] and supported by our unpublished data, lowers ROS levels and may attenuate redox-dependent signaling pathways that normally stimulate the expression of periodically activated cell-cycle genes such as CycB. As a result, CycB levels may be slightly reduced without impairing overall mitotic regulation, keeping mitotic parameters comparable to those observed in the control. While this study integrates cytological observations with selected G2/M markers (CDKA and CycB), further refinement could include additional cell-cycle regulators or direct measurements of CDK activity to better define how Cd and TO modulate the mitotic regulatory network.

**Figure 15:**
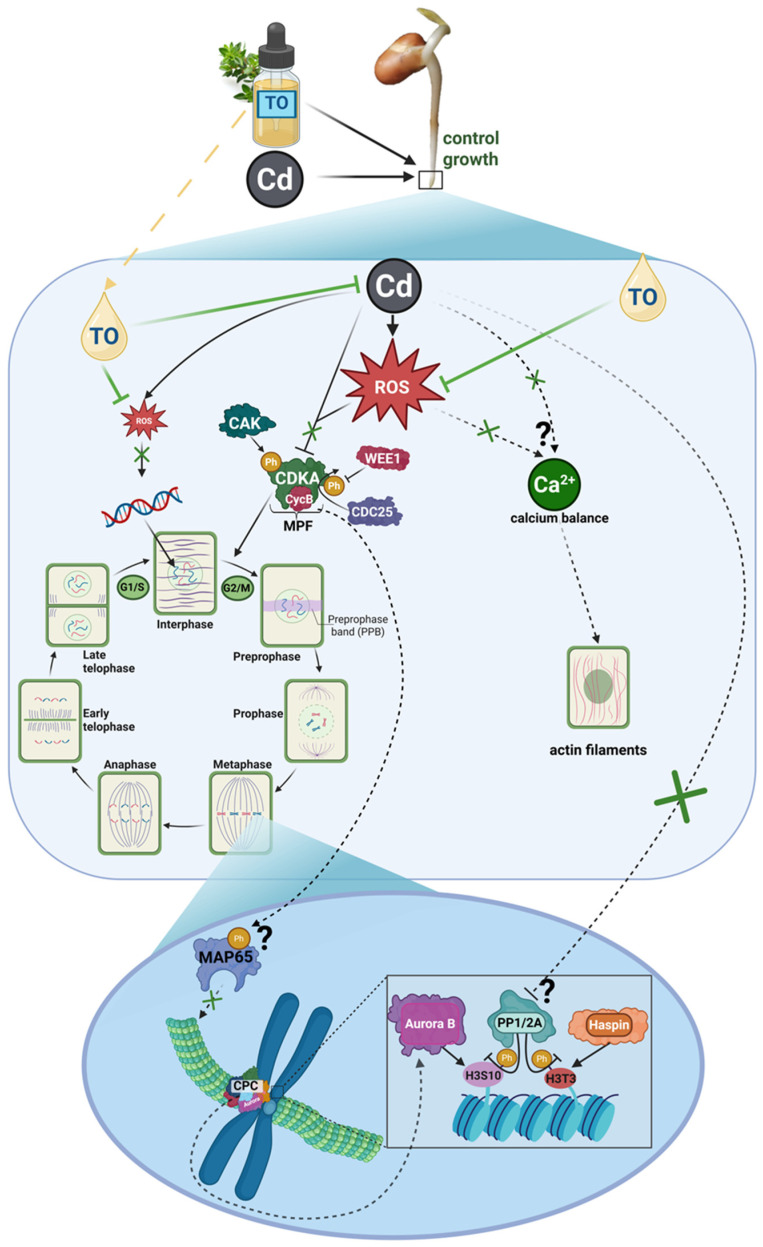
Proposed protective mechanism of thyme oil (TO) against cadmium (Cd)-induced mitotic disturbances. Arrows indicate the direction of processes or signaling pathways; arrows with pointed tips denote activation or enhancement, whereas arrows with blunt (flat) tips denote inhibition or suppression. Solid arrows indicate relationships directly supported by experimental data obtained in our study. Dashed arrows and question marks indicate proposed mechanisms inferred from prior literature and not directly tested here. Cross symbols (X) indicate inhibition or suppression of the indicated processes or interactions. TO treatment is proposed to mitigate Cd-induced oxidative stress, thereby helping to restore calcium homeostasis and proper regulation of CDK–Cyc complexes. This may facilitate proper M-phase Promoting Factor (MPF) activity, enabling inhibitory phosphorylation of MAP65 and preventing excessive microtubule stabilization. TO may also support PP1/PP2A activity, contributing to balanced regulation of mitotic kinases such as Aurora B and Haspin, and thereby maintaining appropriate histone H3 phosphorylation dynamics and chromatin organization. Collectively, the proposed TO-induced changes could increase the mitotic index and promote restoration of root growth in plants exposed to cadmium toxicity. Created in BioRender. Gocek-Szczurtek N. (2026) https://BioRender.com/7jv86za.

TO applied during seedling exposure to CdCl_2_ revealed a clear protective effect against cadmium toxicity in plant cells. Disturbances observed under CdCl_2_ treatment alone were largely reduced or absent in the combined treatment. At the macroscopic level, root growth remained close to the control, and the dynamics of cell division, although not fully restored to control values, were maintained at a relatively high level. The protective action of TO was reflected in the preservation of normal interphase chromatin structure and mitotic chromosome organization, as well as in the maintenance of proper actin filaments and microtubule architecture within the cytoskeleton. This was accompanied by sustained functionality of CDKA–CycB regulatory complexes and by the correct progression of mitosis-related histone H3 phosphorylation (Figure 15).

The collected results indicate that TO exerts a protective effect at a higher organizational level, integrating multiple aspects of cellular organization and cell cycle regulation. This effect may be linked to modulation of redox homeostasis, which could act as a coordinating hub integrating chromatin remodeling, cytoskeletal organization, mitotic enzyme activity, and the execution of epigenetic modifications. This interpretation is consistent with previously reported antioxidant properties of TO, attributed primarily to its main components, thymol, p-cymene, and carvacrol, which have been shown to limit ROS accumulation in various experimental systems [59,135,136,137].

This notion is supported by the study of Dentato et al. [138], who demonstrated that basil extracts, containing among other compounds carvacrol, promoted germination and growth of *Raphanus sativus* seedlings exposed to Cd by enhancing antioxidant enzyme activity, reducing ROS levels, and consequently limiting DNA damage. Consistent observations were reported in our previous publication, in which experiments were conducted in parallel with those presented here and were based on the same experimental system and identical conditions. In that work, Cd-induced ROS overproduction and its significant reduction in the presence of TO were directly demonstrated. Moreover, TO reduced replication stress and DNA damage in *V. faba* interphase cells by limiting oxidative stress [59] and by enhancing the activity of enzymatic and non-enzymatic antioxidant defense systems (unpublished data). Furthermore, TO prevented excessive oxidative stress and stabilized epigenetic modifications and chromatin organization in interphase, supporting transcription, in line with our parallel experiments [60]. In the presence of TO, cadmium-exposed cells did not accumulate excessively in interphase but progressed through it more evenly and entered mitosis, contributing to the maintenance of a substantial proportion of mitotic cells in the meristems, as observed in the present study. However, the population of dividing cells did not reach control levels and remained approximately 50% lower than in the untreated control. This phenomenon can be interpreted in terms of CDKA–CycB hysteresis, according to which the minimal level of CycB and CDKA activity required for entry into mitosis is higher than that needed to maintain mitotic progression. Thus, cells that have already obtained a “mitotic license” may tolerate moderate decreases in CDKA activity and continue dividing, whereas cells that have not yet reached the G2/M decision point do not initiate mitosis under suboptimal complex activity [130,138,139]. In light of our results, although *CycB* transcription in the CdCl_2_ + TO treatment reached or slightly exceeded control levels, the amount of CycB protein, despite being higher than in cells exposed only to cadmium, may still have been insufficient to trigger mitotic entry. This may particularly concern cells that had just completed interphase in a TO-protected yet still cadmium-containing environment and were approaching the G2/M decision point. Consequently, likely only a subset of the population could reach the CDKA–CycB activity threshold required for mitotic entry, especially since CDKA levels (detected by immunofluorescence) did not fully reach those observed in control cells.

With regard to the cells that nevertheless proceeded to division, an antioxidant mechanism of TO action also appears plausible. Based on literature reports, it can be proposed that in a cellular environment protected by TO, compensatory cytoskeletal stabilization mechanisms, such as increased tubulin acetylation or enhanced interactions with MAPs [90,94,97], are not strongly induced. Consequently, as observed in our study, the cytoskeleton does not undergo excessive rigidification or bundling and retains the dynamic properties required for the formation of the karyokinetic and cytokinetic spindle. This supports proper metaphase-to-anaphase transition and maintains proportions of cells at individual mitotic stages comparable to those in the control (Figure 15). Similar restorative effects of antioxidants on ROS-disrupted cytoskeletal organization have also been reported in other experimental models [140,141].

Previous studies conducted in the same experimental system demonstrated that TO reduces ROS levels [59]. This effect, potentially linked to the activation of antioxidant defense mechanisms, may favor the maintenance of PP1/PP2A activity, which, according to literature reports, can be impaired by cadmium-induced oxidative stress [122]. Preservation of these phosphatases supports proper H3T3 phosphorylation dynamics, essential for correct chromosome condensation, interaction with the spindle apparatus, and sister chromatid separation. This interpretation is consistent with literature reports showing that normalization of PP1/PP2A activity restores appropriate histone H3 phosphorylation profiles [142]. Further support for this interpretation comes from studies on other natural antioxidants. For example, literature data indicate that resveratrol protects phosphatases from oxidative inactivation and restores proper protein phosphorylation dynamics in cadmium-exposed neurons [143]. A similar relationship may apply to Aurora B kinase, whose localization and activity are reported to be sensitive to redox disturbances [124,125]. In our study, the restoration of distinct pericentromeric labeling and the disappearance of dispersed signals in the presence of TO suggest that reduced oxidative stress favored the recovery of proper Aurora B localization and the H3S10 phosphorylation pattern characteristic of normal mitosis. In this context, TO may be viewed as a factor supporting mitotic homeostasis by enabling the reversibility of epigenetic changes induced by cadmium-triggered oxidative stress (Figure 15).

Considering the complexity of the cellular response to cadmium stress, the reduction of ROS levels alone likely does not explain the full spectrum of TO’s protective action. Literature data indicate that TO, as well as related essential oils rich in phenolic compounds and sesquiterpenes, can reduce cadmium toxicity and bioavailability in tissues. Ye et al. [144] demonstrated that thymol mitigates cadmium stress in *Nicotiana tabacum* seedlings by modulating glutathione (GSH) and phytochelatin levels, thereby promoting Cd sequestration into bound forms and its redistribution away from the cytoplasm. Similarly, Rahmani et al. [145] and Askari et al. [146] showed in animal models that thyme and clove oils containing caryophyllene markedly reduce Cd accumulation in tissues, accompanied by enhanced antioxidant activity and reduced oxidative stress. A common conclusion from these studies is that induction of endogenous Cd chelation (involving GSH and metallothioneins/phytochelatins) decreases the pool of biologically active Cd within the cell, thereby lowering its cytoplasmic bioavailability. Limiting the availability of free cadmium reduces its capacity to induce oxidative stress, while strengthened antioxidant defenses helps mitigate the effects of the remaining active fraction. Literature reports further indicate that both the reduced oxidative stress and decreased Cd bioavailability may indirectly limit secondary disturbances in calcium homeostasis arising from ROS- and Ca^2+^-dependent signaling pathways [147,148,149]. Stabilization of these cellular processes may support proper cell cycle progression. Consequently, the activity of cyclin-dependent kinase complexes, such as CDKA–CycB, may remain more appropriately regulated [150], potentially enabling correct phosphorylation of microtubule-associated proteins, including MAP65, and supporting proper cytoskeletal organization during mitosis [147,148,149].

In light of the obtained results, TO appears to mitigate the negative effects of Cd exposure in meristematic cells by supporting the preservation of principal elements of proper mitotic progression. This may allow maintenance of reduced yet still effective mitotic activity, reflected in the retention of root growth potential. Although the detailed molecular mechanisms require further investigation, the results indicate a protective role of TO in sustaining cell division under cadmium-induced stress (Figure 15).

## 4. Materials and Methods

### 4.1. Plant Material

Selected based on the appropriate size and color, sterile seeds of *Vicia faba* var. *minor* L. (W. Legutko The Seed Breeding Company in Jutrosin, Poland) were sown in trays lined with moist blotting paper and germinated at a temperature of 20 ± 1 °C in the dark. Four-day-old seedlings with primary roots approximately 2.5 cm long were placed in Petri dishes containing distilled water (control), the emulsifier solution used for essential oil emulsification prepared and handled in the same manner as the TO-containing solution, 175 µM cadmium chloride solution (CdCl_2_), 0.03% (*v*/*v*) emulsified thyme essential oil (TO; Etja, Elbląg, Poland), and CdCl_2_ supplemented with emulsified TO. The composition of the TO was previously characterized by GC-MS [59] and corresponds to a thymol-rich chemotype of *Thymus vulgaris*, with thymol (28.39%), *p*-cymene (23.86%), linalool (8.49%), caryophyllene (4.04%), carvacrol (3.04%), limonene (3.15%), α-pinene (2.91%), γ-terpinene (2.55%), and α-terpineol (2.36%) as the main constituents. All experiments presented in our previous publications, referenced in the Discussion [59,60], in the present study, and in unpublished work assessing the efficiency of antioxidant systems in meristems were performed using the same batch of oil as analyzed in the first work [59] to ensure compositional consistency. The applied CdCl_2_ concentration was determined through a combination of literature analysis and preliminary optimization experiments evaluating doses between 100 and 200 µM [59,60,74,87,151,152]. The final concentration of TO was chosen based on preliminary screening tests (0.01–0.06% *v*/*v*) and supported by earlier reports [59,60]. The results of these preliminary tests, conducted during the optimization phase of the experimental system, evaluating different Cd and TO concentrations, as well as their combined effects, are summarized in Table S1 (Supplementary Materials). Due to the lipophilic nature of the essential oil, an emulsified form was used to obtain a stable and homogeneous dispersion in the aqueous medium and to ensure uniform contact with the biological material. Treatments with non-emulsified oil were excluded from further analyses due to a lack of reproducibility. To distribute TO sufficiently in water, it was emulsified in a mixture of C14-18 and unsaturated C16-18—mono- and di-ethoxylated glycerides and ethoxylated *Brassica napus* oil. The emulsifier mentioned above was mixed with TO in a ratio of 1:4 (*v*/*v*) (resulting in a final emulsifier concentration of 0.0075% *v*/*v*) and vortexed vigorously for 15 min; the emulsifier-only control underwent the same mixing and vortexing procedure. The emulsifier was donated by the Department of Agronomy at Poznań University of Life Sciences. Incubations were performed for 24 h in airtight, tall Petri dishes in the dark. The 24 h incubation period was chosen because it corresponds approximately to the duration of a complete mitotic cycle in root meristematic cells, allowing the detection of cadmium-induced alterations across successive phases of the cell cycle. Primary root meristems were used for analysis. The plant material used in the studies originated from independent germination batches and incubations performed on different days. Seedlings derived from these independent experimental runs were randomly assigned to the respective treatment conditions and served as biological replicates. The number of primary roots examined in each treatment depended on the type of analysis and is given in the relevant methodological subsections, together with detailed information on the number of analyzed cells or nuclei.

### 4.2. Root Length Measurement

Seedlings were measured using ImageJ software ver. 1.54p before and after 24 h incubation of root meristems with water (control), the emulsifier, CdCl_2_, TO, and the combination of CdCl_2_ and TO from the root tip to the hypocotyl. Root length increments were calculated by subtracting the length of the root before treatment from the length of the root after treatment. Overall, 17 seedlings were used for measurements in each experimental series.

### 4.3. Feulgen Staining for Mitotic and Mitotic Phase Indices

Apical parts of primary roots of *V. faba* seedlings were fixed in a mixture of cold absolute ethanol and glacial acetic acid (3:1, *v*/*v*) for 1 h. After rinsing several times with ethanol, the dissected root meristems were rehydrated and hydrolyzed in 4 M HCl for 1 h. Then, after rinsing with distilled water, the roots were placed in Schiff’s reagent (acidified pararosaniline solution; Sigma-Aldrich, St. Louis, MI, USA) for 1 h. After staining, the roots were first rinsed in SO_2_-water and then in distilled water. The cut, apical segments of the roots were then placed in a drop of 45% acetic acid and crushed on microscope slides. The slides were then frozen on dry ice, the coverslips were removed, and the slides were rinsed in 70% ethanol, then left to dry and embedded in Canada balsam. For each experimental series, 5 seedlings were used, and 500 nuclei were analyzed per root, resulting in 2500 Feulgen DNA-stained cells analyzed per series. The mitotic index (IM) value was calculated as the ratio of dividing cells to all meristematic cells × 100%. Phase index (IF) values were calculated as the ratio of the number of cells in a specific phase of mitosis to all mitotic cells × 100%. Photographic documentation was performed using an Eclipse E-600 microscope and a DS-Fi1 digital camera (Nikon, Tokyo, Japan).

### 4.4. Immunocytochemical Staining of Microtubules (β-Tubulin) and Actin Filaments

Root apical fragments (3 mm) were fixed in 4% (*w*/*v*) paraformaldehyde buffered with MTSB (50 mM PIPES, 5 mM EGTA, 5 mM MgSO4, pH 7.0) for 45 min, and then rinsed twice in PBS and transferred to the citrate-buffered mixture containing 2.5% (*w*/*v*) pectinase, 2.5% (*w*/*v*) cellulase and 2.5% (*w*/*v*) pectolyase (Sigma-Aldrich); pH 5.0; 40 °C) for 45 min. After rinsing with cold PBS (4 °C), root meristems were crushed on Super Frost Plus slides (Menzel-Gläser, Thermo Fisher Scientific, Waltham, MA, USA) in a drop of cold PBS. After freezing with dry ice, coverslips were removed, slides were washed with distilled water, and air-dried. Macerated cells were permeabilized with 0.5% (*v*/*v*) Triton X-100 for 15 min, then treated with blocking buffer (8% BSA and 0.1% Triton X-100, PBS; 20 °C) for 50 min. Slides were then rinsed with PBS and incubated overnight in a humid atmosphere (4 °C) with mouse monoclonal anti-β-tubulin antibodies (1:750, Sigma-Aldrich) and monoclonal actin antibodies (Sigma-Aldrich). All antibodies were dissolved in the antibody dilution buffer (1% *w*/*v* BSA, 0.3% *v*/*v* Triton X-100, PBS). The slides were then washed in PBS and incubated with the secondary Alexa Fluor 488-conjugated anti-rabbit IgG (1:500, Sigma-Aldrich) dissolved in the antibody dilution buffer (1% *w*/*v* BSA, 0.3% *v*/*v* Triton X-100, PBS) at 25 °C for 90 min. Nuclear DNA was stained with 5 µM DAPI (Sigma-Aldrich) for 15 min, and then the slides were washed in PBS. The specimens were mounted in PBS/glycerol mixture (9:1) containing 2.5% 1,4-diazabicyclo[2.2.2]octane (DABCO). For each experimental series, 3 root apical fragments were used.

### 4.5. Immunocytochemical Detection of CDKA Kinase and Phosphorylated H3 Histones (Thr3 and Ser10)

Root meristems from *V. faba* cuttings were fixed in PBS-buffered 4% (*w*/*v*) paraformaldehyde for 45 min, washed with PBS, and placed in the citrate-buffered mixture containing 2.5% (*w*/*v*) pectinase, 2.5% (*w*/*v*) cellulase, and 2.5% (*w*/*v*) pectolyase (Sigma-Aldrich) (pH 5.0; 40 °C) for 45 min. After washing with PBS and distilled water, root meristems were squashed onto slides, air dried, and pre-treated with PBS-buffered 8% BSA and 4% Triton X-100 (Sigma-Aldrich) for 50 min (20 °C). Then, slides were incubated with rabbit polyclonal anti-CDC2 antibody (1:500; Agrisera, Vännäs, Sweden), recognizing the conserved plant CDKA homolog; rabbit polyclonal anti-H3S10Ph antibody (1:500; Sigma-Aldrich); and rabbit polyclonal anti-H3T3Ph antibody (1:500; Sigma-Aldrich). All antibodies were dissolved in PBS containing 1% BSA (*w*/*v*) and 0.3% Triton X-100 (*v*/*v*). Incubations were carried out overnight in a humid atmosphere at 4 °C. Following rinsing with PBS, slides were incubated with secondary goat anti-rabbit conjugated to Alexa Fluor^®^ 488 antibody (1:500; Cell Signaling Technology, Danvers, MA, USA) dissolved in PBS at 20 °C for 90 min. Slides were counterstained with 5 µM DAPI (Sigma-Aldrich) at 20 °C for 15 min., washed with PBS, and embedded in PBS/glycerol mixture (9:1) containing 2.5% 1,4-diazabicyclo[2.2.2]octane (DABCO). For immunocytochemical analyses, five seedlings per treatment were used for CDKA and H3S10Ph detection, and three seedlings per treatment for H3T3Ph detection. Within each root meristem, fluorescence intensity was measured in multiple nuclei: 20 nuclei per root for CDKA (100 nuclei per treatment), 10 nuclei per root for H3S10Ph (50 nuclei per treatment), and 10 nuclei per root for H3T3Ph (30 nuclei per treatment).

### 4.6. Western Blotting of Kinase CDKA and CycB

Proteins were extracted from apical parts of primary roots of *V. faba* with the use of Pierce™ Plant Total Protein Extraction Kit (Thermo Fisher Scientific, Waltham, MA, USA) supplemented with Protease Inhibitor Cocktail (Sigma-Aldrich). Protein concentration was spectrophotometrically measured using Pierce BCA Protein Assay Kit (Thermo Fisher Scientific). The extracts were fractionated on NuPAGE^®^ Novex^®^ 4–12% Bis-Tris gel (Thermo Fisher Scientific) and then blotted onto polyvinylidene fluoride (PVDF) membrane, 0.2-μm pore size (Thermo Fisher Scientific). Membrane blocking, antibody incubation, and chromogenic detection were performed using the WesternBreeze Chromogenic Kit, anti-rabbit (Thermo Fisher Scientific), according to the manufacturer’s protocol. CDKA protein kinase was detected using polyclonal Anti-CDC2 antibodies (Agrisera, Vännäs, Sweden) diluted 1:3000, recognizing the conserved plant CDKA homolog. CycB was detected using polyclonal Anti-CycB antibodies (Agrisera) diluted 1:2500. Actin was detected using monoclonal actin antibodies (Sigma-Aldrich) diluted 1:4000. Membrane blocking, antibody incubation, and chromogenic detection were performed using WesternBreeze Chromogenic Kit, anti-rabbit (Thermo Fisher Scientific) according to the manufacturer’s protocol. The chromogenic signal was developed for 60 min for CDKA and CycB and 30 min for Actin. PVDF membranes were imaged with the ProXima 2750 imaging system (Isogen Life Sciences, De Meern, The Netherlands). Quantitative microdensitometric evaluation of the membranes was carried out using GelAnalyzer version 23.1.1 (www.gelanalyzer.com, accessed on 1 March 2025; Istvan Lazar Jr., PhD, and Istvan Lazar Sr., PhD). Following image import, the software automatically identified lane baselines and protein bands. Band intensities were determined from lane profiles by integrating the selected peaks and correcting the values by subtracting the corresponding baseline.

For Western blot analyses, each biological replicate corresponded to an independent incubation experiment, from which apical regions of 5 primary roots were pooled to obtain sufficient protein for extraction. The blots presented in the figures are representative of 3 replicate experiments showing consistent patterns. Densitometric quantification was performed for each biological replicate (*n* = 3) using GelAnalyzer 23.1.1 (available at www.gelanalyzer.com (accessed on 1 March 2025) by Istvan Lazar Jr., PhD, and Istvan Lazar Sr., PhD, and the obtained values were used for statistical comparisons between treatments.

### 4.7. Gene Expression Analysis

To assess gene expression, five excised roots were snap-frozen in liquid nitrogen and ground into a fine powder using a mortar and pestle. A total of 200 mg of this powder was used for RNA extraction with the NucleoSpin RNA Plant and Fungi Mini Kit (Macherey-Nagel, Düren, Germany), following the manufacturer’s instructions. RNA concentration and purity were measured using a BioDrop Duo spectrophotometer (BioDrop Ltd., Cambridge, UK), and all samples showed A260/A280 ratios below 2.0. The integrity of RNA was confirmed by running 1 µg of each sample on a 1% agarose gel prepared in 1X Tris-acetate-EDTA (TAE) buffer containing 1 mg·mL^−1^ ethidium bromide at 110 V for 1.5 h at room temperature. Distinct ribosomal RNA bands were observed, and no evidence of degradation was detected.

Prior to cDNA synthesis, genomic DNA was removed using RQ1 RNase-Free DNase (Promega, Madison, WI, USA) using 3 µg RNA for each sample. Reverse transcription was carried out using 2 µg of RNA with Oligo(dT)18 primers and SuperScript™ III Reverse Transcriptase (Thermo Fisher Scientific), according to the manufacturer’s protocol. The obtained cDNA was diluted 100X with nuclease-free water and stored at −20 °C until further use.

Quantitative real-time PCR (qPCR) was performed with PowerTrack™ SYBR Green Master Mix (Thermo Fisher Scientific) using 1 ng of cDNA per 10 µL reaction in four technical replicates. Primer concentrations were adjusted to 300 nM, and the annealing/extension temperature was set to 61 °C on a RotorGene Q 5-plex HRM instrument (QIAGEN, Hilden, Germany). Melting curve analysis was conducted to confirm amplification specificity. Relative expression levels were calculated using the 2^−ΔΔCt^ method [153] and normalized to the reference gene cyclophilin (CYP2) [154]. Data are presented as log_2_(fold change), corresponding to −ΔΔCt values. Primers targeting coding sequences (CDS) were designed using Primer3 (available online: https://primer3.ut.ee/, accessed on 1 March 2025) [155] and validated in silico against *V. faba* cv. ‘Hedin/2’ v1 CDS using BLAST (Galaxy platform, IPK Gatersleben, Germany; https://galaxy-web.ipk-gatersleben.de, accessed on 1 March 2025). The primer sequences were as follows:

#### 4.7.1. *CycB*

Forward:5′-GGCTGCTGGTAGAAGTGTGT-3′

Reverse:5′-TGGCATTTATATCGGGCTGTGA-3′

Amplicon size: 176 bp

#### 4.7.2. *CYP2* (Reference Gene)

Forward: 5′-TGCCGATGTCACTCCCAGAA-3′

Reverse: 5′-CAGCGAACTTGGAACCGTAGA-3′

For gene expression analyses, each biological replicate corresponded to an independent incubation experiment, from which five excised primary roots were pooled to obtain sufficient material for RNA extraction. Reverse transcription and qPCR analyses were performed independently for each biological replicate (*n* = 3). Each qPCR reaction was conducted in four technical replicates, and the mean Ct value obtained for a given biological replicate was used for further calculations of relative expression. Statistical comparisons between treatments were performed using the values obtained for the biological replicates.

### 4.8. Microscopic Measurements, Observations and Analyses

Observations were performed with a Nikon Eclipse E600W fluorescence microscope (Nikon, Tokyo, Japan) featuring phase-contrast optics, a U2 filter (UVB light; λ = 340–380 nm) for DAPI and a B2 filter (blue light; λ = 465–496 nm) for Alexa Fluor^®^ 488. All images in each experimental series of a given experiment, using the specific antibodies, were captured at identical integration times with a DS-Fi1 CCD camera (Nikon, Tokyo, Japan). Under these conditions, negative control samples—incubated only with the secondary antibody conjugated to Alexa Fluor^®^ 488, without the primary antibody—did not produce a detectable signal, appearing completely dark in the images. Quantitative analyses and fluorescence intensity (FI) measurements were performed in ImageJ software ver. 1.54p after conversion of the color images to grayscale and were expressed in arbitrary units [a.u.] as the mean pixel value (p.v.) covering the range from 0 (dark) to 255 (white) according to the methods described [156,157]. Standard background subtraction was applied by subtracting the average signal from areas lacking specific fluorescence. In microscopy-based analyses, independent biological replicates consisted of individual seedlings (primary roots) obtained from separate germination batches and independent incubations performed on different days. Depending on the analysis, three to five independent roots were analyzed per treatment. Within each biological replicate, fluorescence intensity was measured in multiple nuclei within the root meristem. Statistical analyses were performed using independent biological replicates (individual roots), while measurements from multiple nuclei within each root were treated as technical observations.

### 4.9. Statistical Analysis

Data were analyzed using GraphPad Prism version 10 software (GraphPad Software, Boston, MA, USA). Before selecting the appropriate statistical test, data normality was assessed using the Shapiro–Wilk test. For datasets meeting the assumptions of normality, one-way analysis of variance (ANOVA) was performed, followed by Tukey’s post hoc test to evaluate differences between groups. For datasets not meeting normality assumptions, the non-parametric Kruskal–Wallis test with appropriate post hoc analysis was applied. In data presentation, variables with a normal distribution are reported as mean ± standard deviation (SD), whereas variables not meeting normality assumptions are presented as median with confidence interval (CI). In all analyses, the significance level was set at *p*  <  0.05. Statistical significance in figures is indicated with asterisks (**** *p* < 0.0001, *** *p* < 0.001, ** *p* < 0.01, * *p* < 0.05).

## 5. Conclusions

In light of the obtained results, TO demonstrates a clear protective potential toward meristematic cells of primary roots of *V. faba* subjected to the toxic effects of cadmium (CdCl_2_) at a concentration of 175 µM during a 24 h incubation. TO, widely described in the literature as a compound with antioxidant properties, when applied at a concentration of 0.03% (*v*/*v*), reversed (to control or near-control levels) disturbances that can be associated with elevated ROS levels typically accompanying cadmium stress.

In the presence of TO, despite the continued presence of cadmium, the control level of the constitutively transcribed CDKA protein was maintained, and high levels of periodically cell-cycle-regulated CycB transcripts and protein were restored; both had been reduced in cells treated with cadmium alone. The maintenance of active CDKA–CycB complexes likely supported proper phosphorylation of microtubule-associated proteins (MAPs), which in turn may have limited excessive microtubule bundling and stabilization, allowing proper spindle organization during karyokinesis and cytokinesis. At the same time, the proper organization of actin filaments was re-established. Moreover, the restoration of the correct mitotic phosphorylation pattern of histone H3 (T3Ph and S10Ph) points to a preserved balance between the activities of the kinases (Haspin and Aurora B) and phosphatases (PP1/PP2A) responsible for these modifications. Such a state favored the reversal of excessive mitotic chromosome condensation, proper recruitment of the chromosomal passenger complex (CPC) in association with Aurora B kinase, and correct spindle microtubule attachment, successful metaphase-to-anaphase transition and accurate sister chromatid segregation, thereby re-establishing proportions of cells at individual mitotic stages similar to those observed in the control. As a result, the tenfold cadmium-induced decrease in the mitotic index was partially reversed, reaching approximately 50% of the control value. These changes were accompanied by primary root growth comparable to that of the control.

An interpretation referring to the antioxidant action of TO is consistent with earlier observations obtained in the same model, where TO alleviated symptoms of oxidative stress in interphase cells, reduced replication stress and the level of DNA damage, stabilized epigenetic modifications and interphase chromatin organization, and supported the maintenance of transcriptional activity while preventing interphase arrest. These effects could have additionally contributed to maintaining, as observed in the present study, a pool of cells capable of entering mitosis.

To the best of our knowledge, this study provides the first evidence that TO may exert a protective effect on the mitotic apparatus of plant meristematic cells exposed to cadmium stress. The obtained data indicate that TO may act as a natural factor supporting plant tolerance to heavy metal stress and could serve as a potential component of strategies aimed at protecting plant cells from the toxic effects of cadmium.

It should be noted, however, that the study was conducted using a single model species and a single exposure duration, which naturally limits the generalizability of the findings. At the same time, this approach allowed for a detailed, multi-level analysis of meristematic cell responses to cadmium stress and an evaluation of the protective effects of TO under controlled conditions. In future studies, it would be advisable to include varied exposure times and concentrations, as well as to extend the analyses to other plant species, which would help better define the dynamics of cellular responses and the universality of the observed mechanisms. Additionally, complementing cytological methods with biochemical analyses, assessing the activity of antioxidant systems, glutathione metabolism, and phytochelatin synthesis, would enable a more comprehensive confirmation of the mechanisms proposed based on the observed cellular effects. In this context, the present work not only documents the protective effect of TO on meristematic cells exposed to cadmium stress but also provides a basis for more in-depth mechanistic studies on the use of biologically active natural compounds to modulate plant responses to environmental stress. From a broader perspective, natural products such as TO, characterized by relatively low toxicity and biodegradability, are increasingly explored as complements to synthetic agrochemicals. Essential oil-based formulations have been proposed for agricultural applications in forms such as foliar sprays, soil amendments, or controlled-release systems [158,159]. However, it should be emphasized that the present study was conducted under controlled laboratory conditions using root meristem cells, and verification of such applications will require further soil-based and field-scale investigations.

## Data Availability

The original contributions presented in this study are included in the article/Supplementary Material. Further inquiries can be directed to the corresponding author.

## Supplementary Materials

The following supporting information can be downloaded at: https://www.mdpi.com/article/10.3390/ijms27062798/s1.

## Abbreviations

The following abbreviations are used in this manuscript:

APC/C	Anaphase-promoting complex/cyclosome
CAK	CDK-activating kinase
Cd	Cadmium
Cdc2	Cell division control 2
CDC25	Cell division cycle 25 phosphatase
CDKs	Cyclin-dependent kinases
CKIs	Cyclin-dependent kinase inhibitors
CMTs	Cortical microtubules
CPC	Chromosomal passenger complex
Cyc	Cyclin
MPF	M-phase-promoting factor
PP1-2A, B, C	Protein Phosphatase 1-2A, B, C
PPB	Preprophase band
ROS	Reactive oxygen species
SAC	Spindle assembly checkpoint
TO	Thyme oil
γ-TuRC	γ-tubulin ring complex
