# Supplementary material for: Thyme Oil Alleviates Cadmium-Induced Disturbances in Mitotic Activity, Cytoskeletal Organization and H3T3/H3S10 Phosphorylation in Vicia faba
Source: Int J Mol Sci. 2026 Mar 19;27(6):2798. doi: 10.3390/ijms27062798 (PMC13026290; doi:10.3390/ijms27062798)
Supplement: Supplementary file 1 [file ijms-27-02798-s001.zip › ijms-4191421-supplementary.pdf]

International Journal of
Molecular Sciences

Table S1. Preliminary screening of $CdCl_2$ and thyme essential oil (TO) concentrations used for selecting the experimental conditions in *Vicia faba* root meristem cells.

Treatment	Concentration	Mitotic index (% ± SD)	% of control	Interpretation
**Control**	**–**	**5.92 ± 1.67**	**100**	**Normal mitotic activity**
$CdCl_2$	100 µM	4.57 ± 1.1	77	Mild inhibition of mitosis
$CdCl_2$	150 µM	2.2 ± 0.7	38	Strong inhibition (used in previous studies [1,2])
**$CdCl_2$**	**175 µM**	**0.54 ± 0.2**	**9**	**Strong but sublethal mitotic inhibition - selected (used in previous studies [3,4])**
$CdCl_2$	200 µM	0.2 ± 0.05	3.4	Severe inhibition; poor meristem/cell condition
TO	0.01%	6.0 ± 1.1	101	No effect on mitosis
**TO**	**0.03%**	**5.6 ± 1.9**	**94**	**Comparable to control - selected**
TO	0.06%	5.1 ± 1.2	86	Mild inhibition of mitotic activity
$CdCl_2$ + TO	175 µM + 0.01%	1.97 ± 0.5	33	Partial recovery of mitotic activity
**$CdCl_2$ + TO**	**175 µM + 0.03%**	**3.3 ± 0.68**	**56**	**Strong mitigation of Cd-induced mitotic inhibition - selected**
$CdCl_2$ + TO	175 µM + 0.06%	2.89 ± 0.97	48.7	Mitigation of Cd-induced mitotic inhibition

References:

1. Żabka, A.; Winnicki, K.; Polit, J.T.; Wróblewski, M.; Maszewski, J. Cadmium (II)-Induced Oxidative Stress Results in Replication Stress and Epigenetic Modifications in Root Meristem Cell Nuclei of *Vicia faba*. *Cells* **2021**, *10*, 640, doi:10.3390/cells10030640.

2. Żabka, A.; Gocek, N.; Winnicki, K.; Szczeblewski, P.; Laskowski, T.; Polit, J.T. Changes in Epigenetic Patterns Related to DNA Replication in *Vicia faba* Root Meristem Cells under Cadmium-Induced Stress Conditions. *Cells* **2021**, *10*, 3409, doi:10.3390/cells10123409.

3. Gocek-Szczurtek, N.; Żabka, A.; Wróblewski, M.; Kukula-Koch, W.; Szczeblewski, P.; Polit, J.T. Thyme Oil Mitigates Cadmium-Induced Oxidative and Genotoxic Stress in *Vicia faba* Root Meristem Cells under in vitro Conditions. *Sci Rep* **2025**, *15*, 38689, doi:10.1038/s41598-025-22588-w.

4. Gocek-Szczurtek, N.; Żabka, A.; Wróblewski, M.; Polit, J.T. Stabilization of the MAPK–Epigenetic Signaling Axis Underlies the Protective Effect of Thyme Oil Against Cadmium Stress in Root Meristem Cells of *Vicia faba*. *IJMS* **2025**, *27*, 208, doi:10.3390/ijms27010208.

Disclaimer/Publisher's Note: The statements, opinions and data contained in all publications are solely those of the individual author(s) and contributor(s) and not of MDPI and/or the editor(s). MDPI and/or the editor(s) disclaim responsibility for any injury to people or property resulting from any ideas, methods, instructions or products referred to in the content.
